# Supplementary figures and images for: Improved ANAP incorporation and VCF analysis reveal details of P2X7 current facilitation and a limited conformational interplay between ATP binding and the intracellular ballast domain
Source: eLife. 2023 Jan 4;12:e82479. doi: 10.7554/eLife.82479 (PMC9859053; doi:10.7554/eLife.82479)

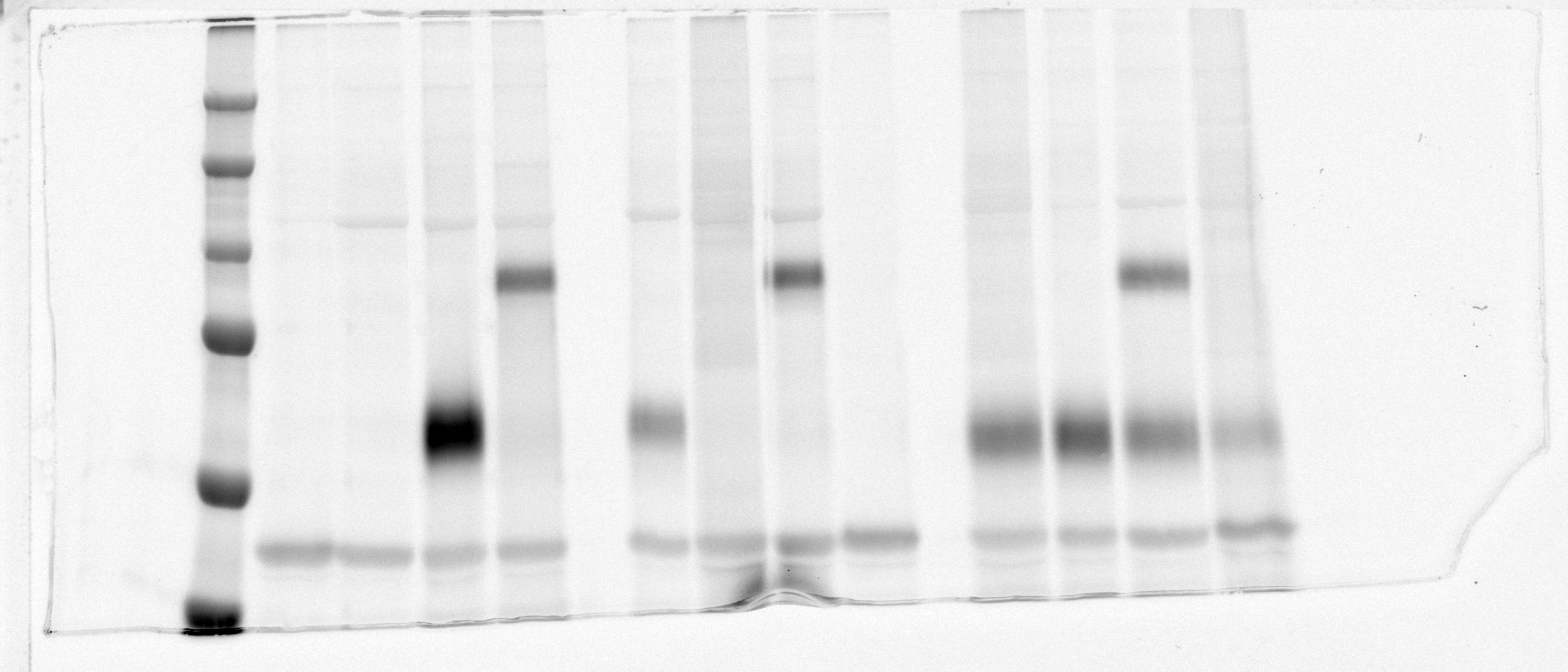

Supplement: Figure 1—source data 1. [file elife-82479-fig1-data1.zip › Figure-1_source-data-1/Gel1.tif]

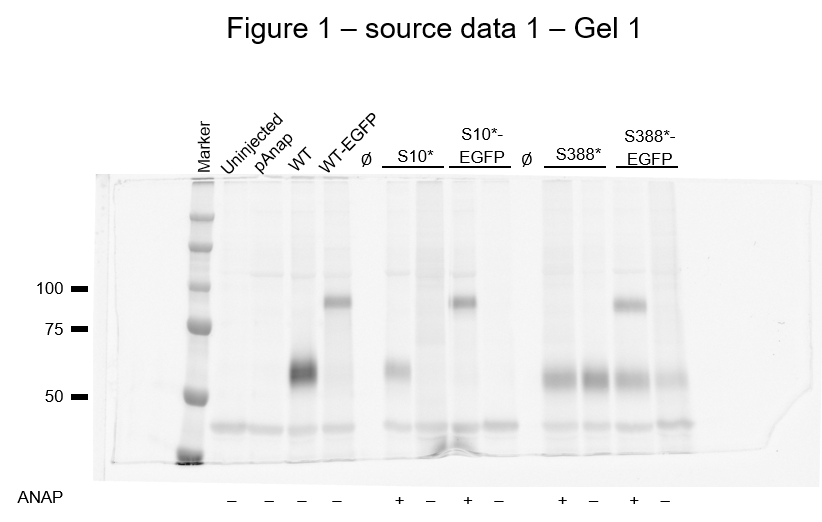

Supplement: Figure 1—source data 1. [file elife-82479-fig1-data1.zip › Figure-1_source-data-1/Gel1_uncropped.png]

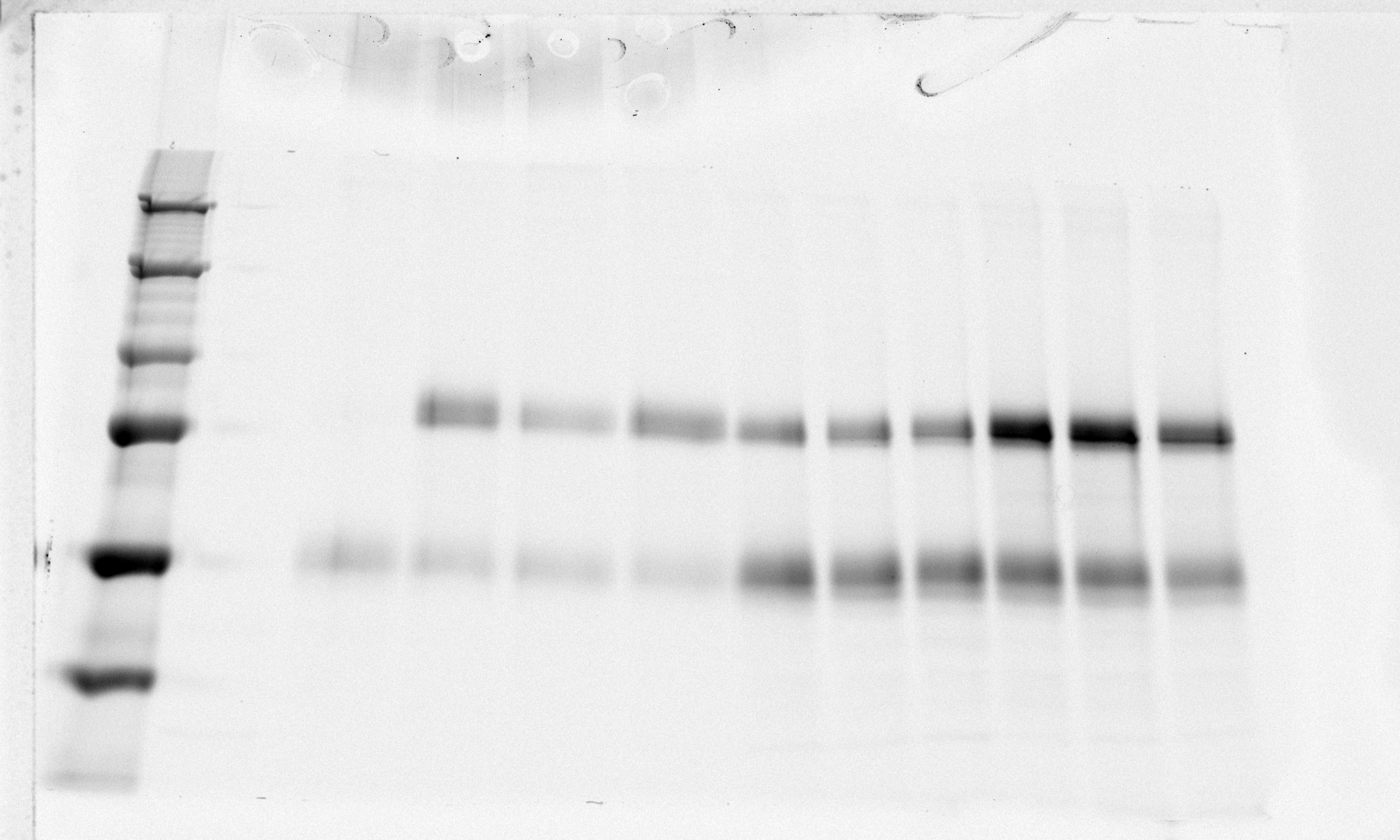

Supplement: Figure 1—source data 2. [file elife-82479-fig1-data2.zip › Figure-1_source-data-2/Gel2.tif]

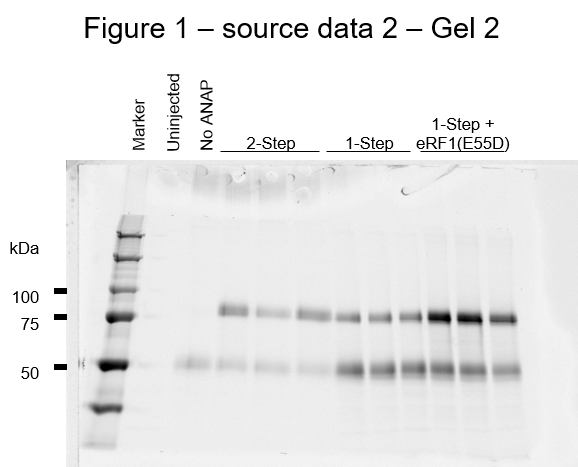

Supplement: Figure 1—source data 2. [file elife-82479-fig1-data2.zip › Figure-1_source-data-2/Gel2_uncropped.png]

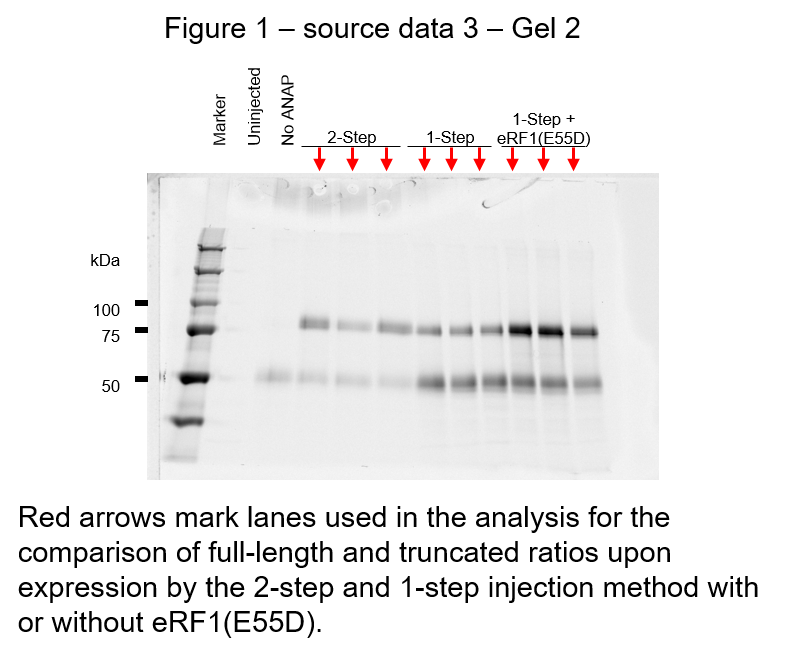

Supplement: Figure 1—source data 3. [file elife-82479-fig1-data3.zip › Figure-1_source-data-3/Gel2/Gel2uncropped.png]

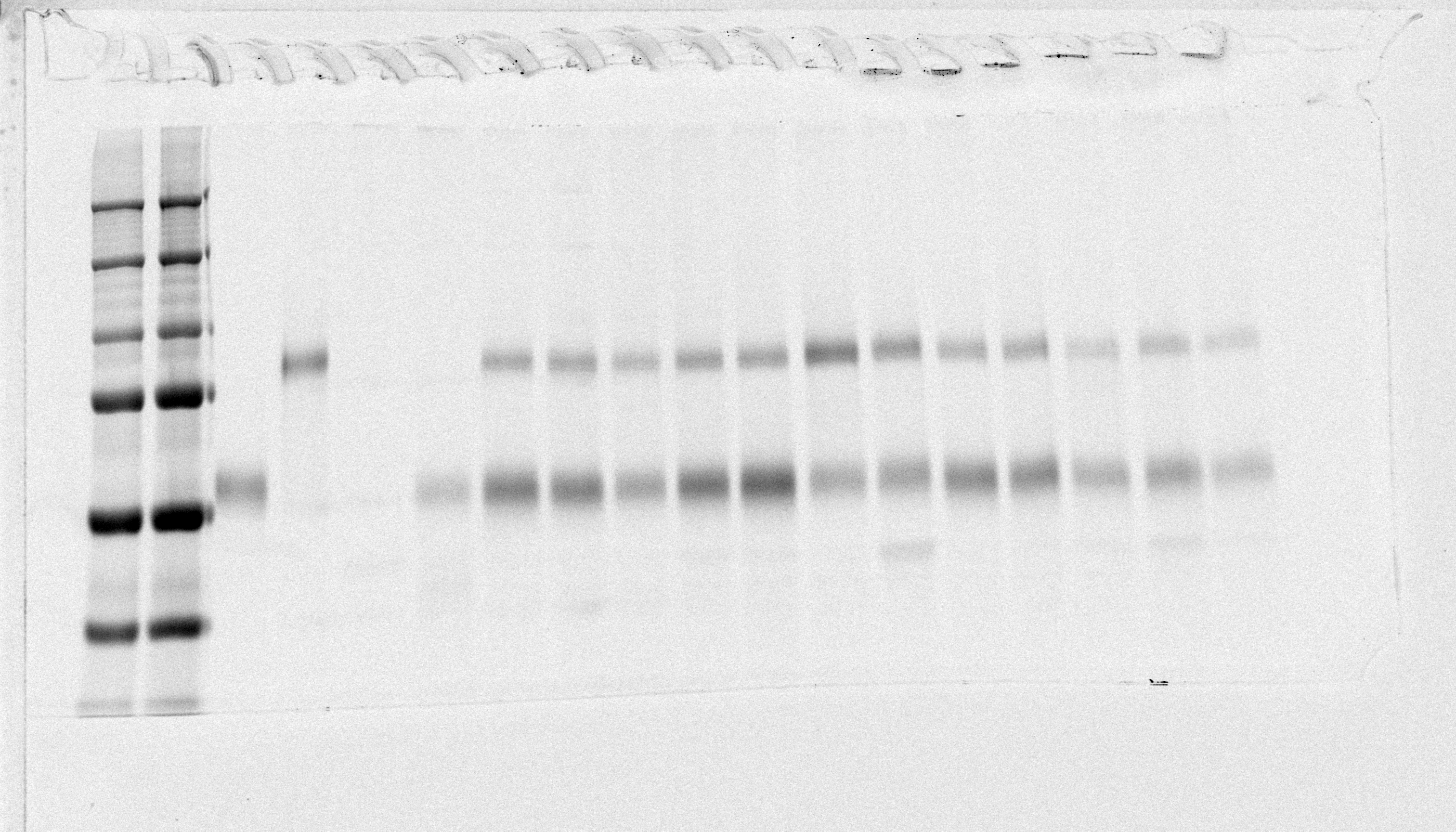

Supplement: Figure 1—source data 3. [file elife-82479-fig1-data3.zip › Figure-1_source-data-3/Gel3/Gel3.gel]

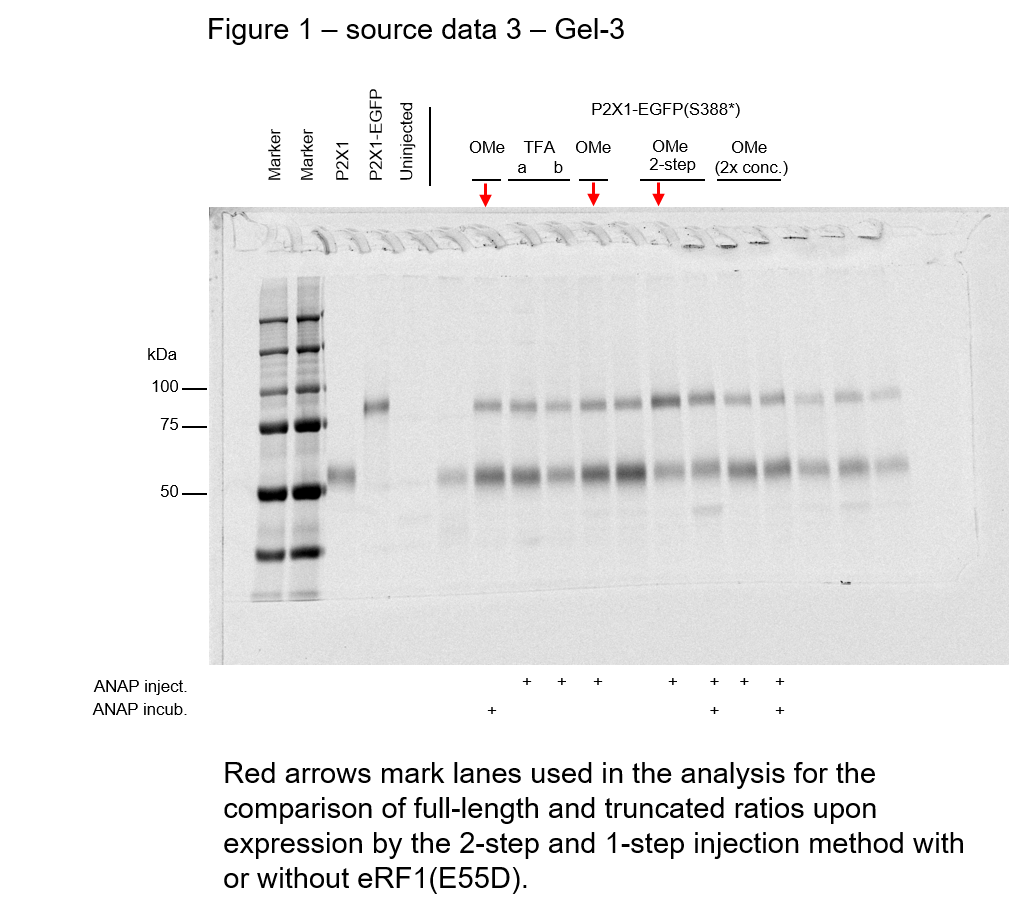

Supplement: Figure 1—source data 3. [file elife-82479-fig1-data3.zip › Figure-1_source-data-3/Gel3/Gel3uncropped.png]

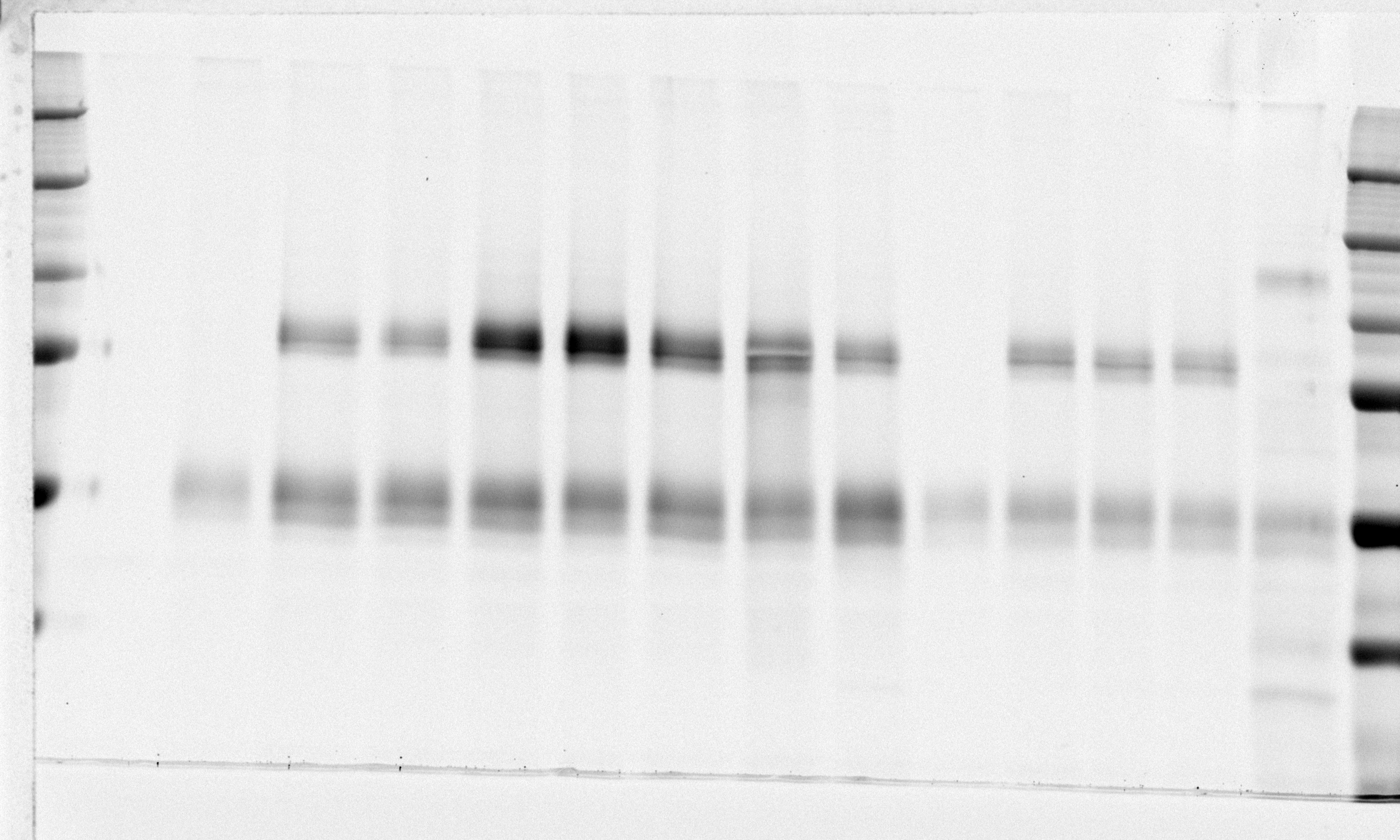

Supplement: Figure 1—source data 3. [file elife-82479-fig1-data3.zip › Figure-1_source-data-3/Gel4/Gel4.gel]

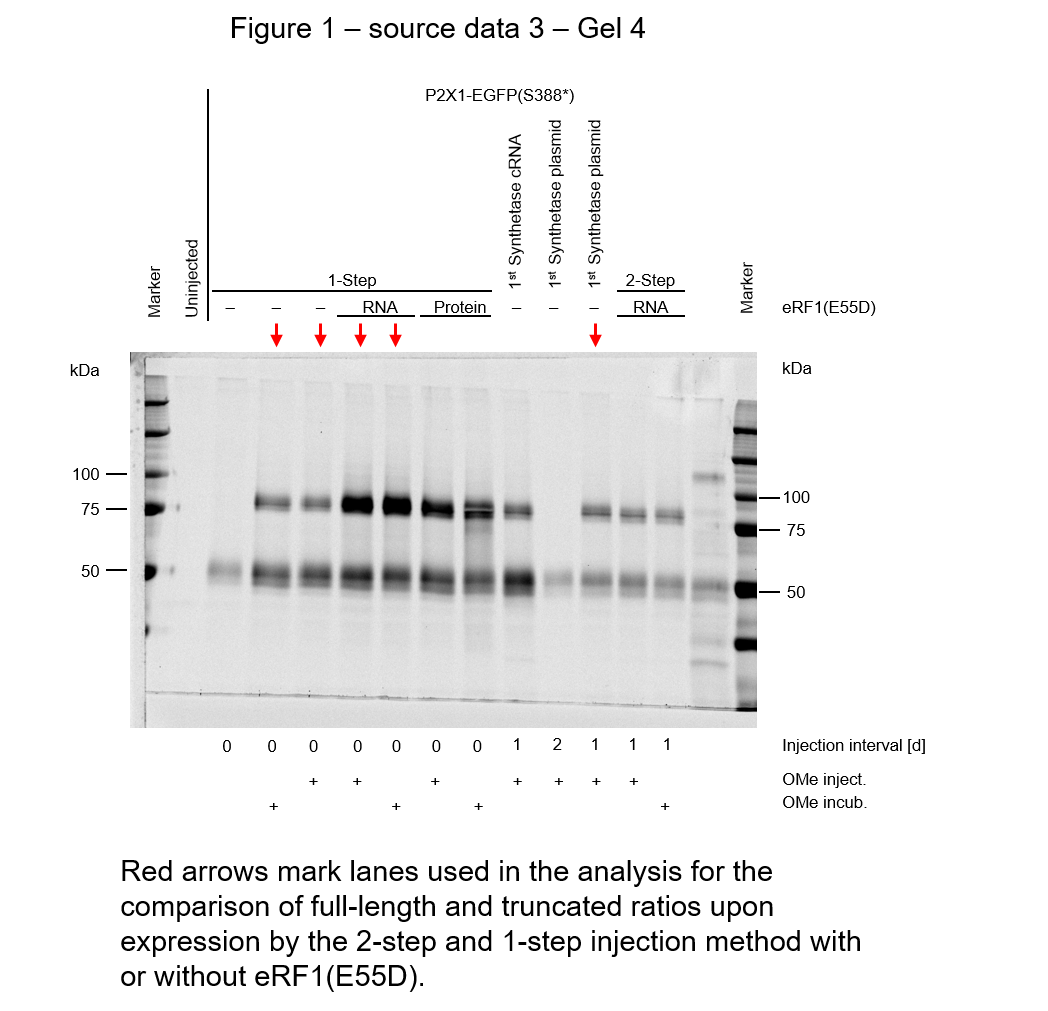

Supplement: Figure 1—source data 3. [file elife-82479-fig1-data3.zip › Figure-1_source-data-3/Gel4/Gel4uncropped.png]

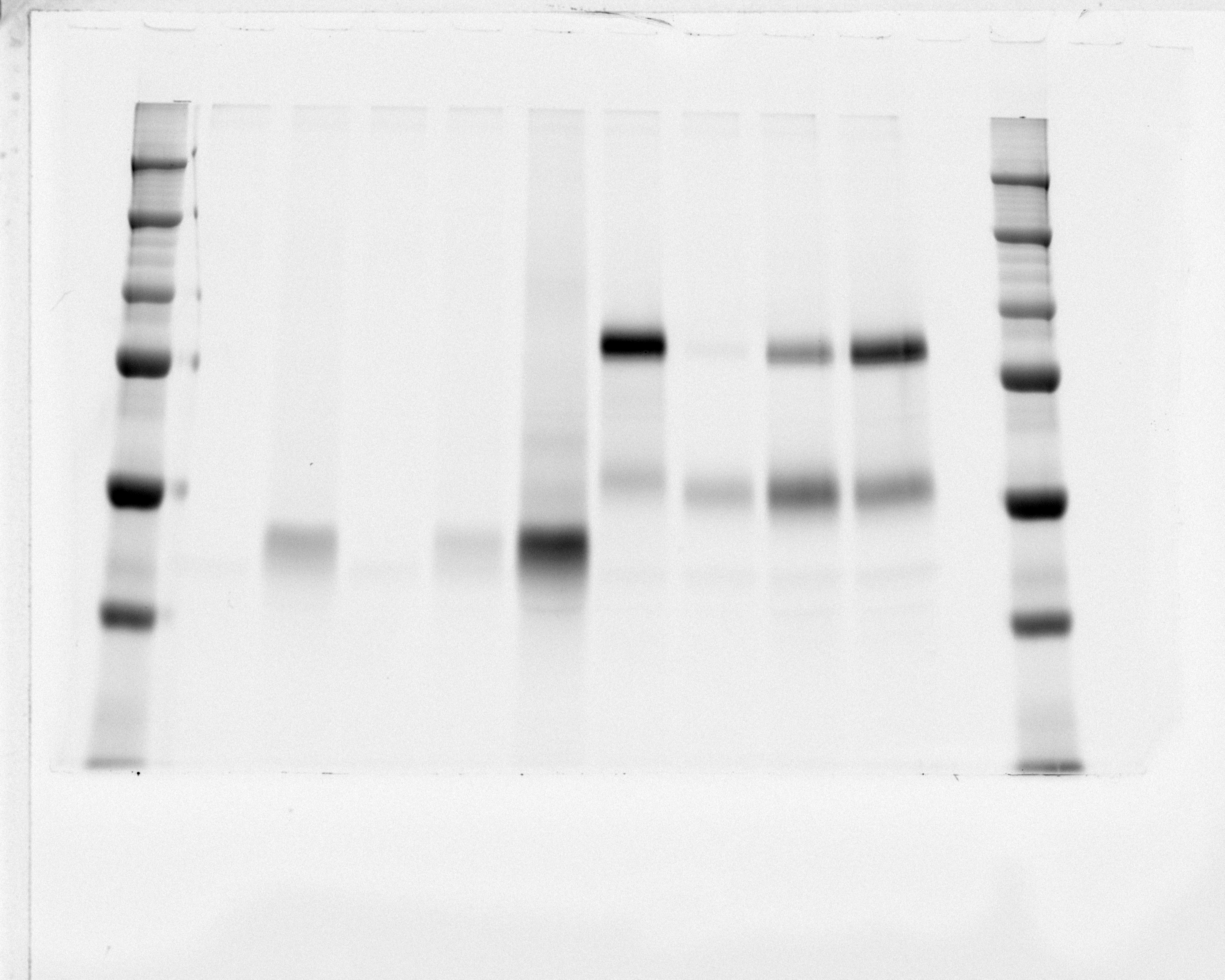

Supplement: Figure 1—source data 3. [file elife-82479-fig1-data3.zip › Figure-1_source-data-3/Gel5/Gel5.gel]

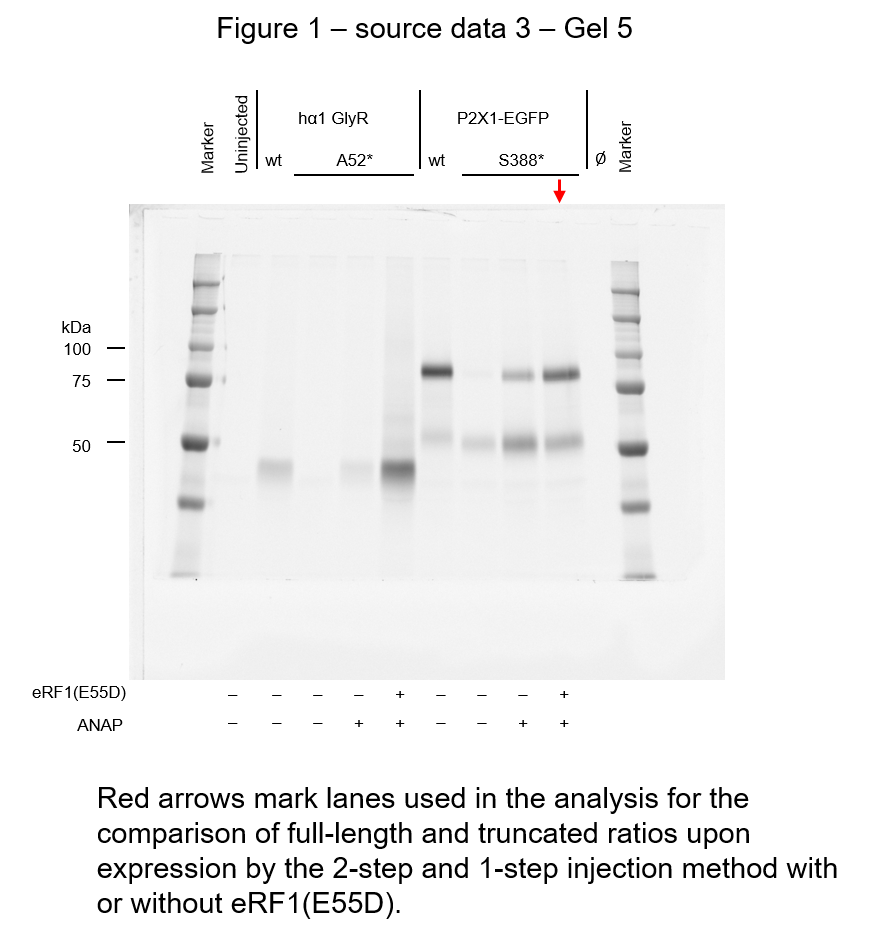

Supplement: Figure 1—source data 3. [file elife-82479-fig1-data3.zip › Figure-1_source-data-3/Gel5/Gel5uncropped.png]

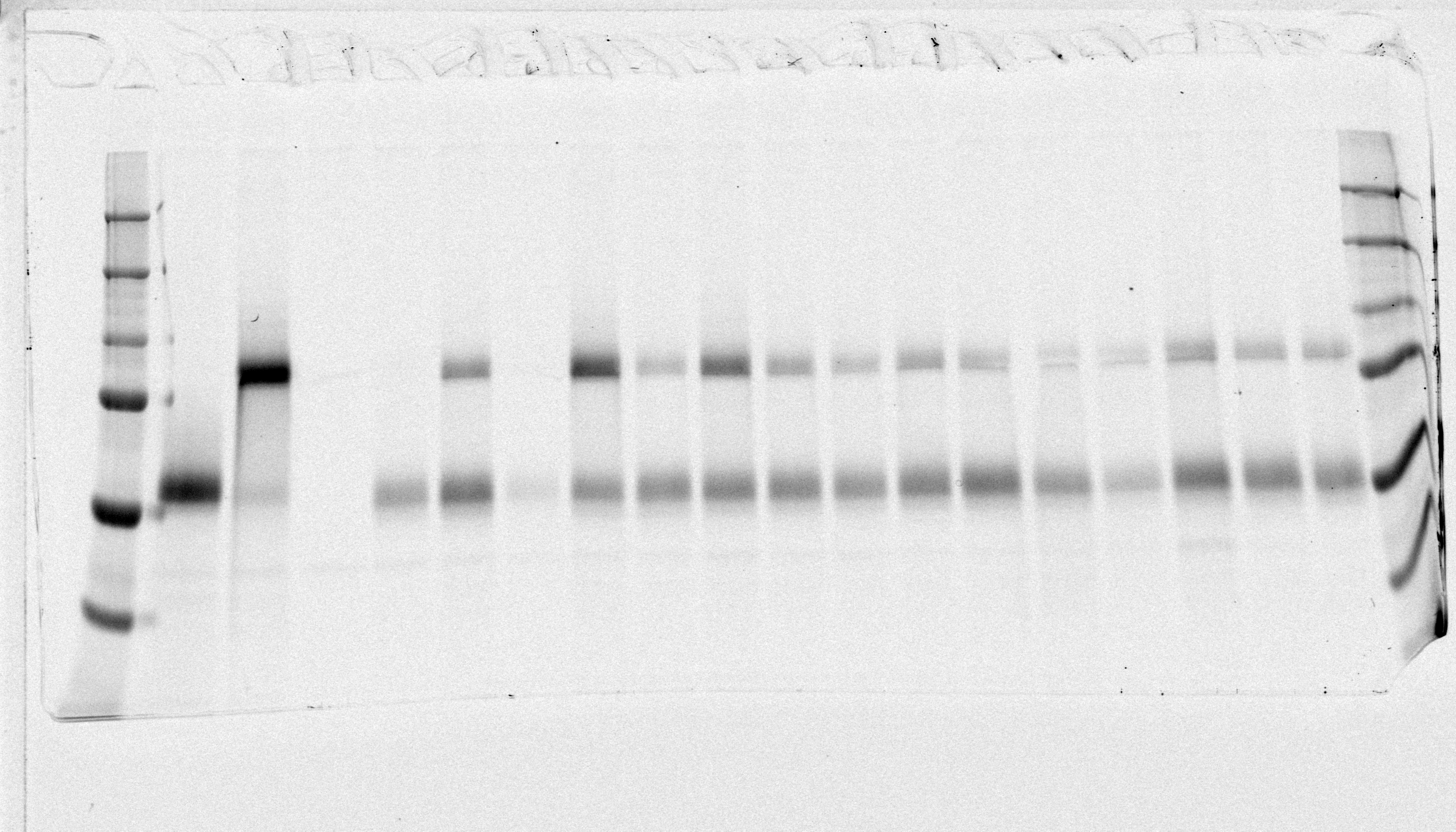

Supplement: Figure 1—source data 4. [file elife-82479-fig1-data4.zip › Figure-1_source-data-4/GelA/GelA.gel]

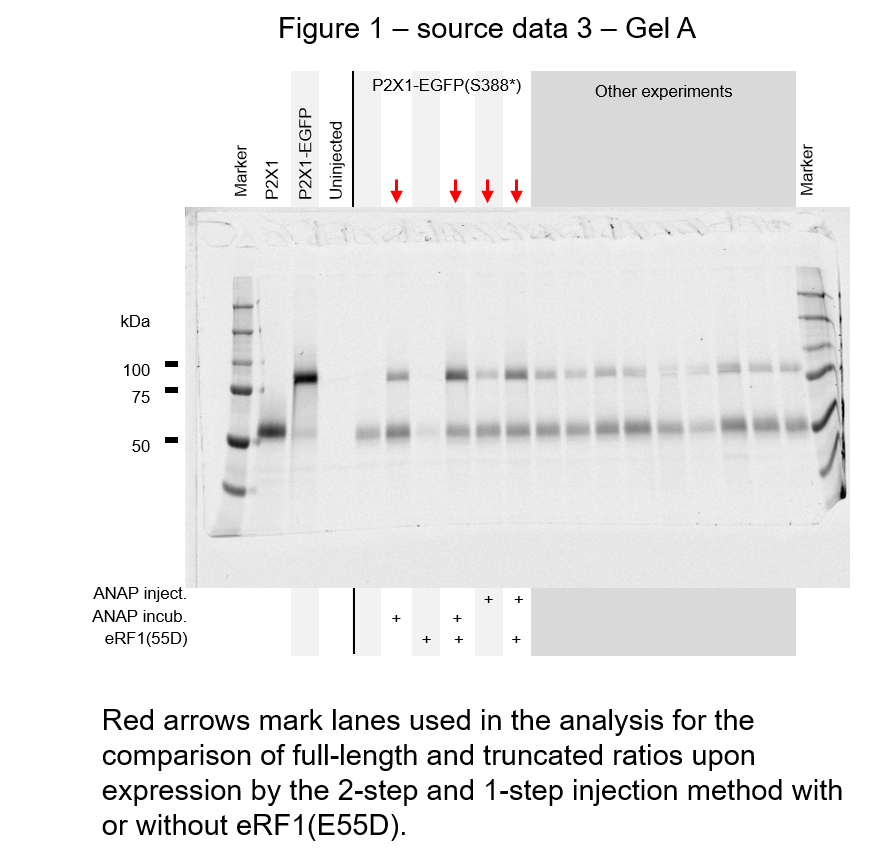

Supplement: Figure 1—source data 4. [file elife-82479-fig1-data4.zip › Figure-1_source-data-4/GelA/GelAuncropped.png]

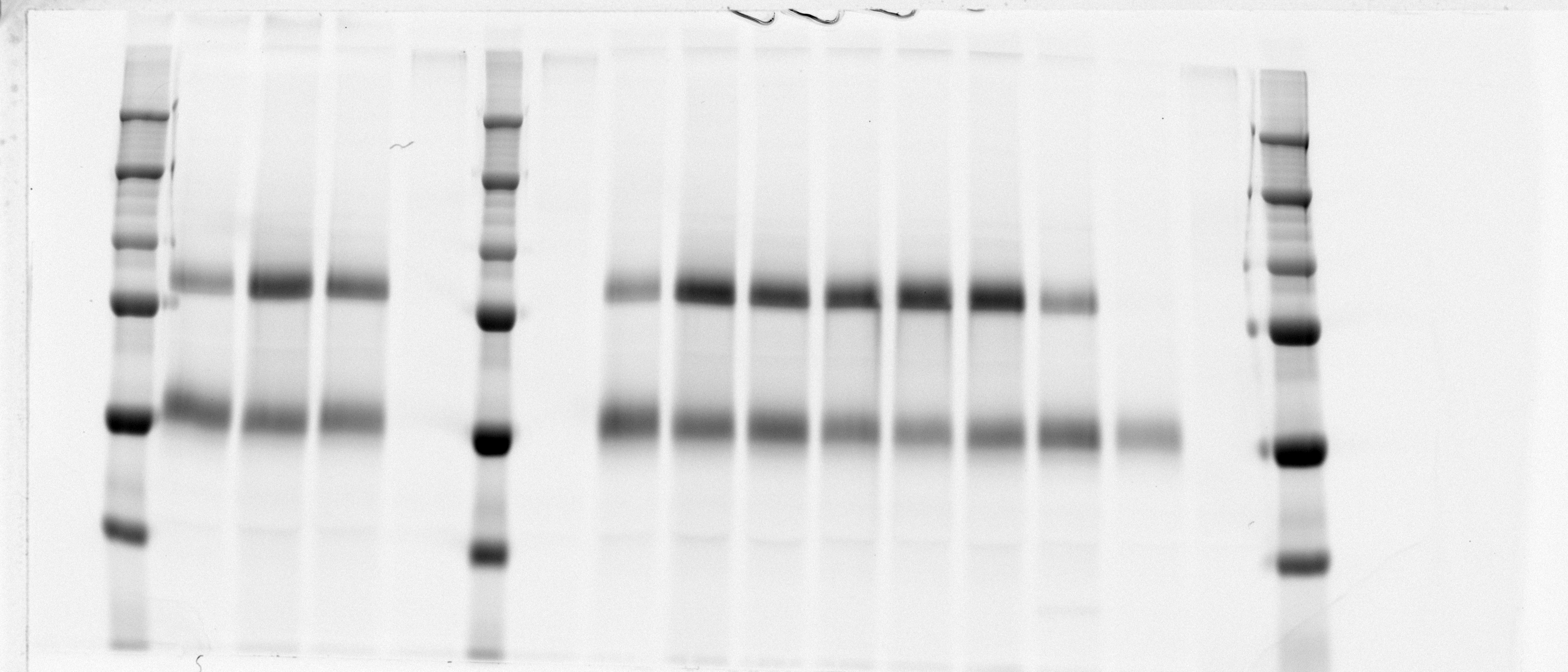

Supplement: Figure 1—source data 4. [file elife-82479-fig1-data4.zip › Figure-1_source-data-4/GelB/GelB.gel]

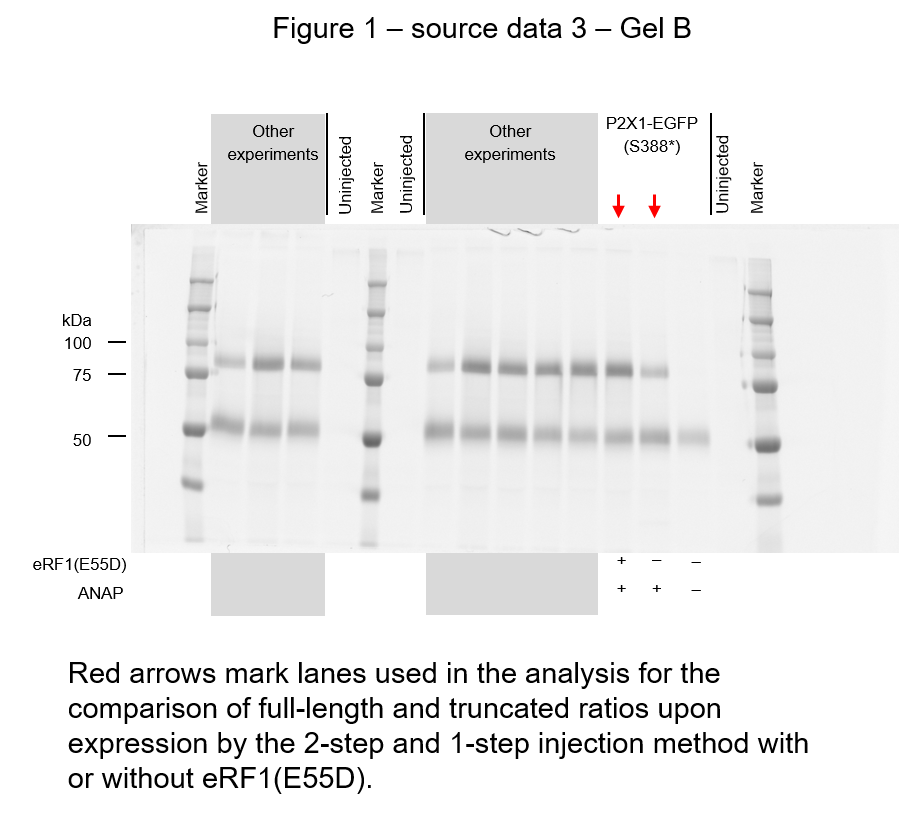

Supplement: Figure 1—source data 4. [file elife-82479-fig1-data4.zip › Figure-1_source-data-4/GelB/GelBuncropped.png]

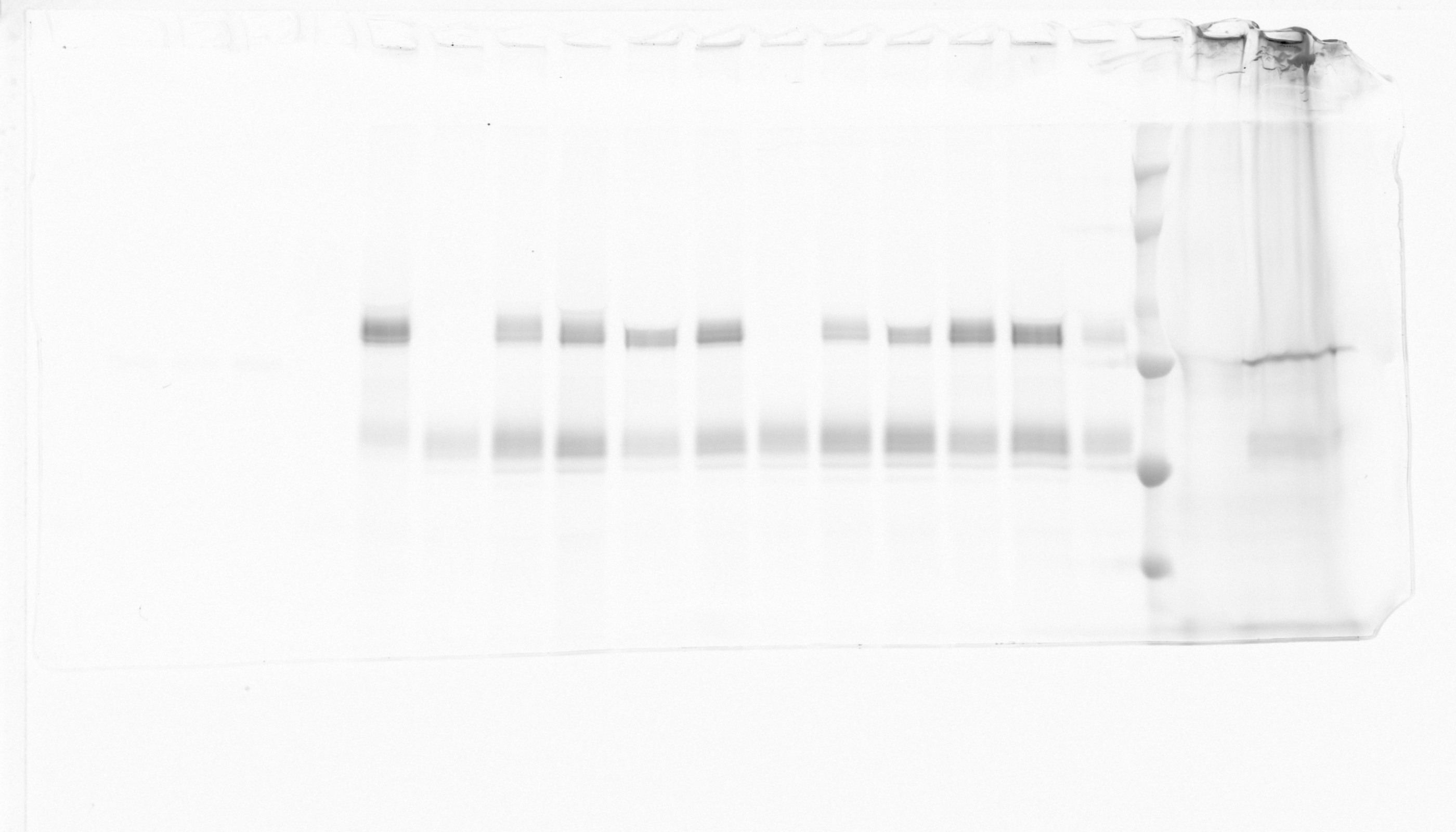

Supplement: Figure 1—source data 4. [file elife-82479-fig1-data4.zip › Figure-1_source-data-4/GelC/GelC.gel]

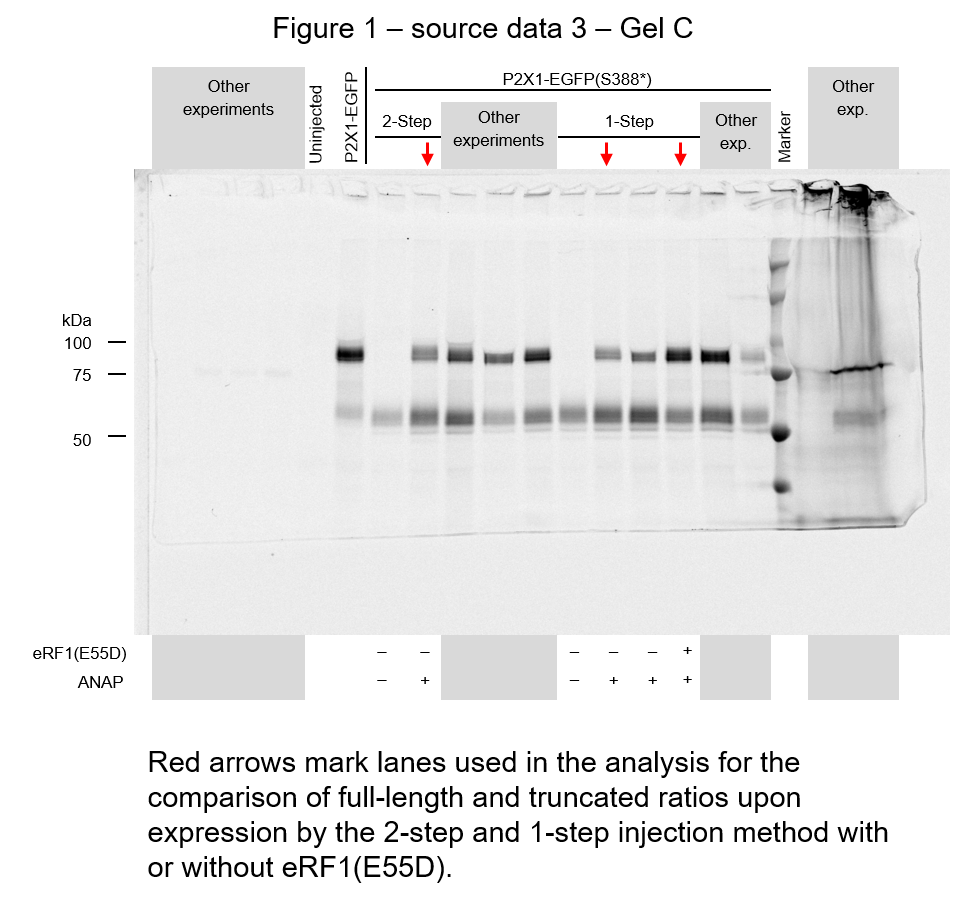

Supplement: Figure 1—source data 4. [file elife-82479-fig1-data4.zip › Figure-1_source-data-4/GelC/GelCuncropped.png]

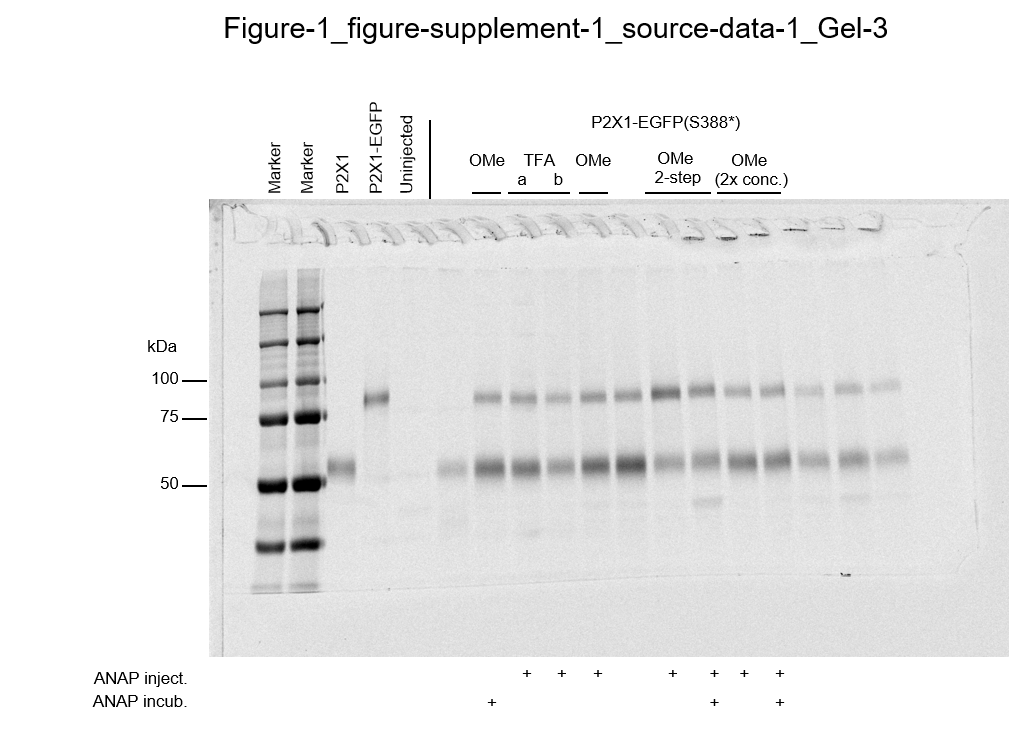

Supplement: Figure 1—figure supplement 1—source data 1. [file elife-82479-fig1-figsupp1-data1.zip › Figure-1_figure-supplement-1_source-data-1/Gel3uncropped.png]

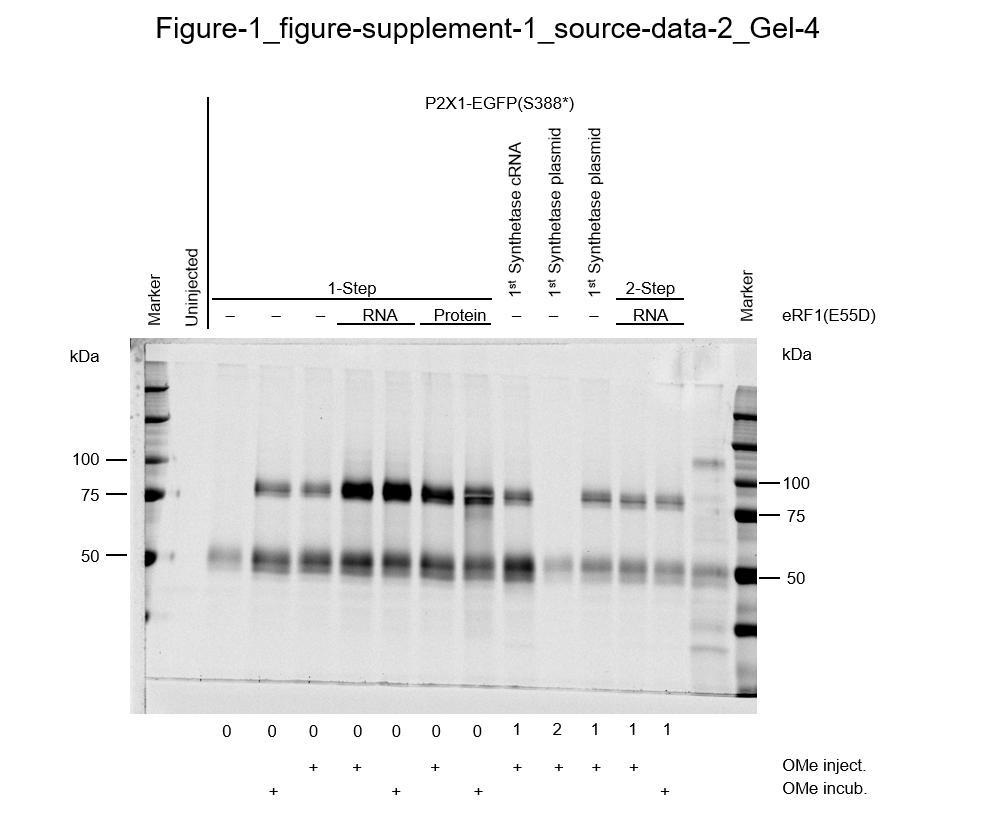

Supplement: Figure 1—figure supplement 1—source data 2. [file elife-82479-fig1-figsupp1-data2.zip › Figure-1_figure-supplement-1_source-data-2/Gel4uncropped.png]

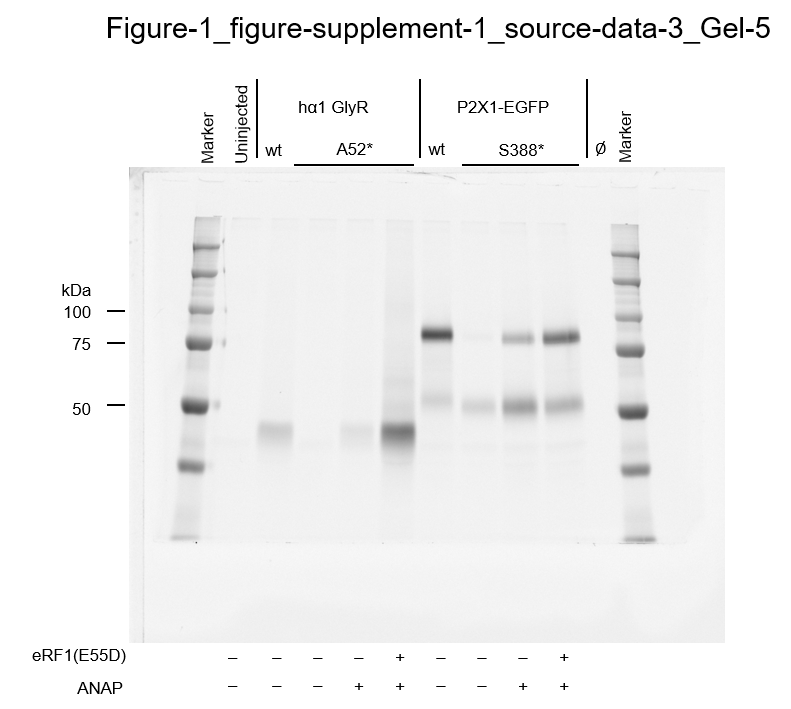

Supplement: Figure 1—figure supplement 1—source data 3. [file elife-82479-fig1-figsupp1-data3.zip › Figure-1_figure-supplement-1_source-data-3/Gel5uncropped.png]

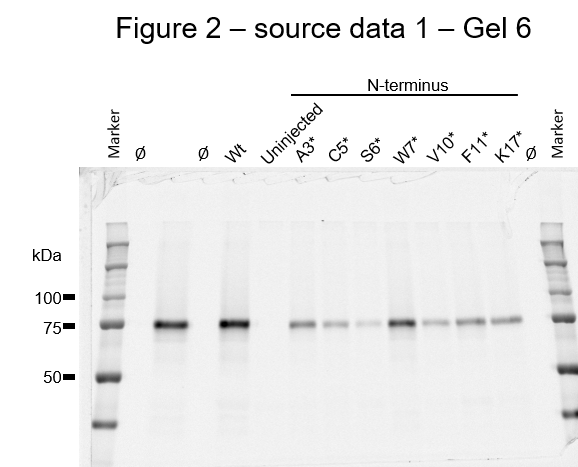

Supplement: Figure 2—source data 1. [file elife-82479-fig2-data1.zip › Figure-2_source-data-1/Gel6uncropped.png]

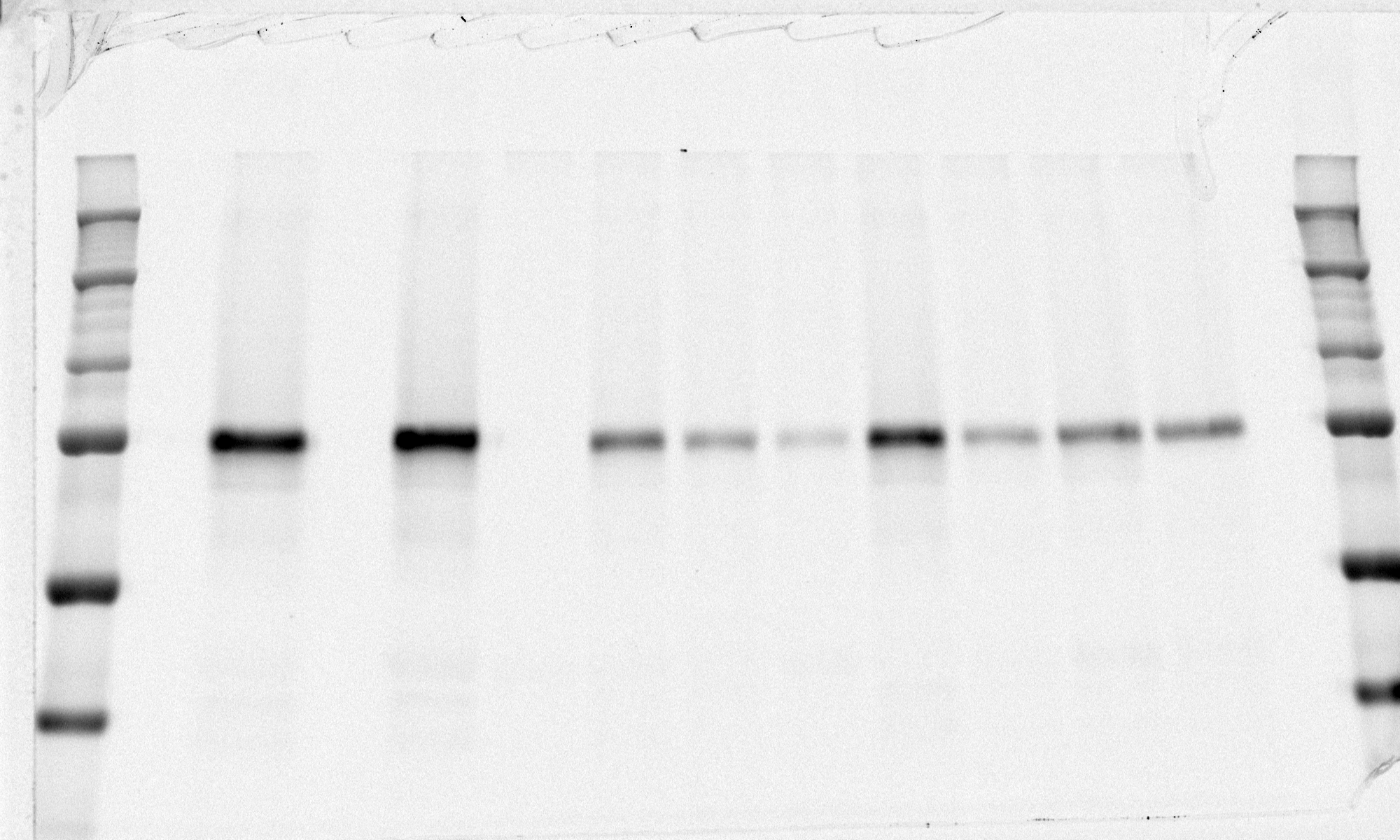

Supplement: Figure 2—source data 1. [file elife-82479-fig2-data1.zip › Figure-2_source-data-1/Gel6.tif]

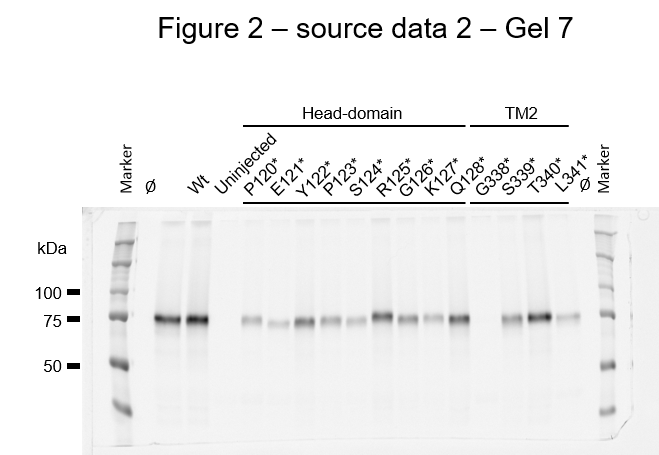

Supplement: Figure 2—source data 2. [file elife-82479-fig2-data2.zip › Figure-2_source-data-2/Gel7uncropped.png]

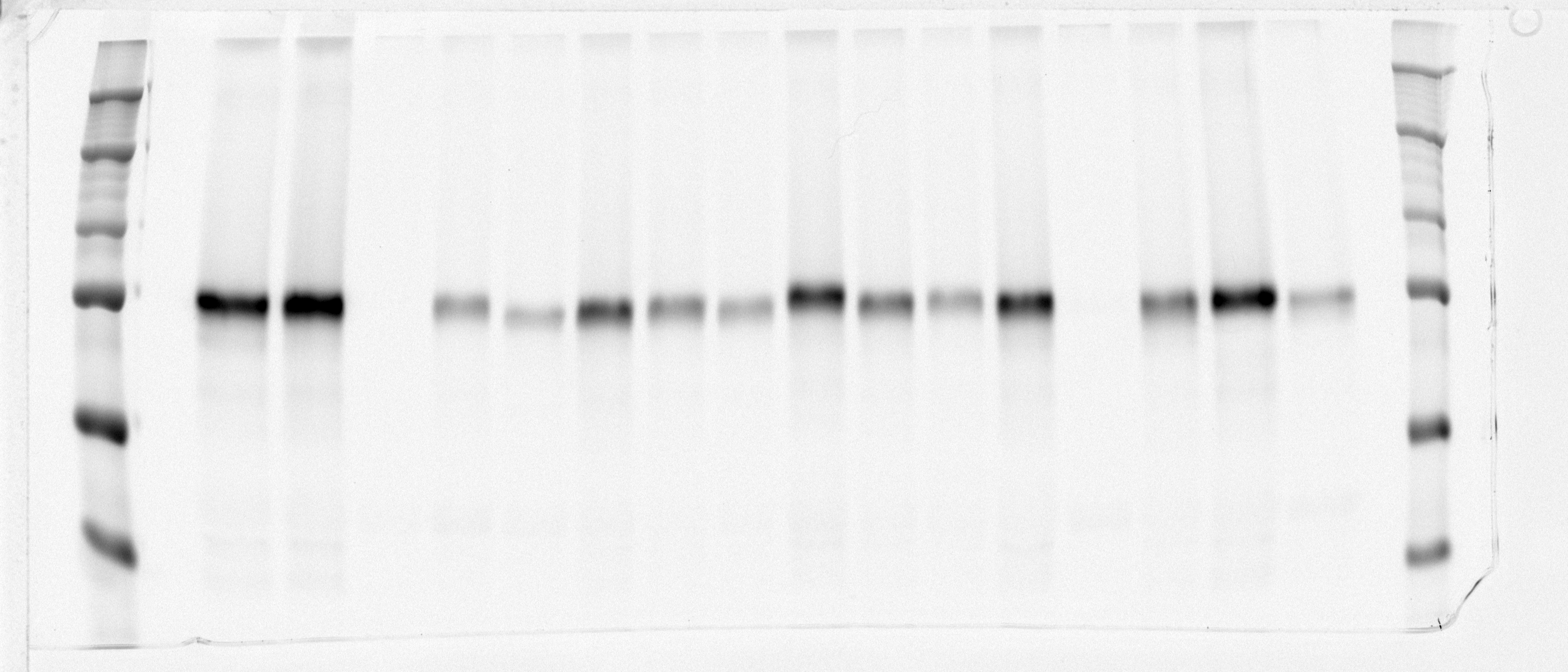

Supplement: Figure 2—source data 2. [file elife-82479-fig2-data2.zip › Figure-2_source-data-2/Gel7.tif]

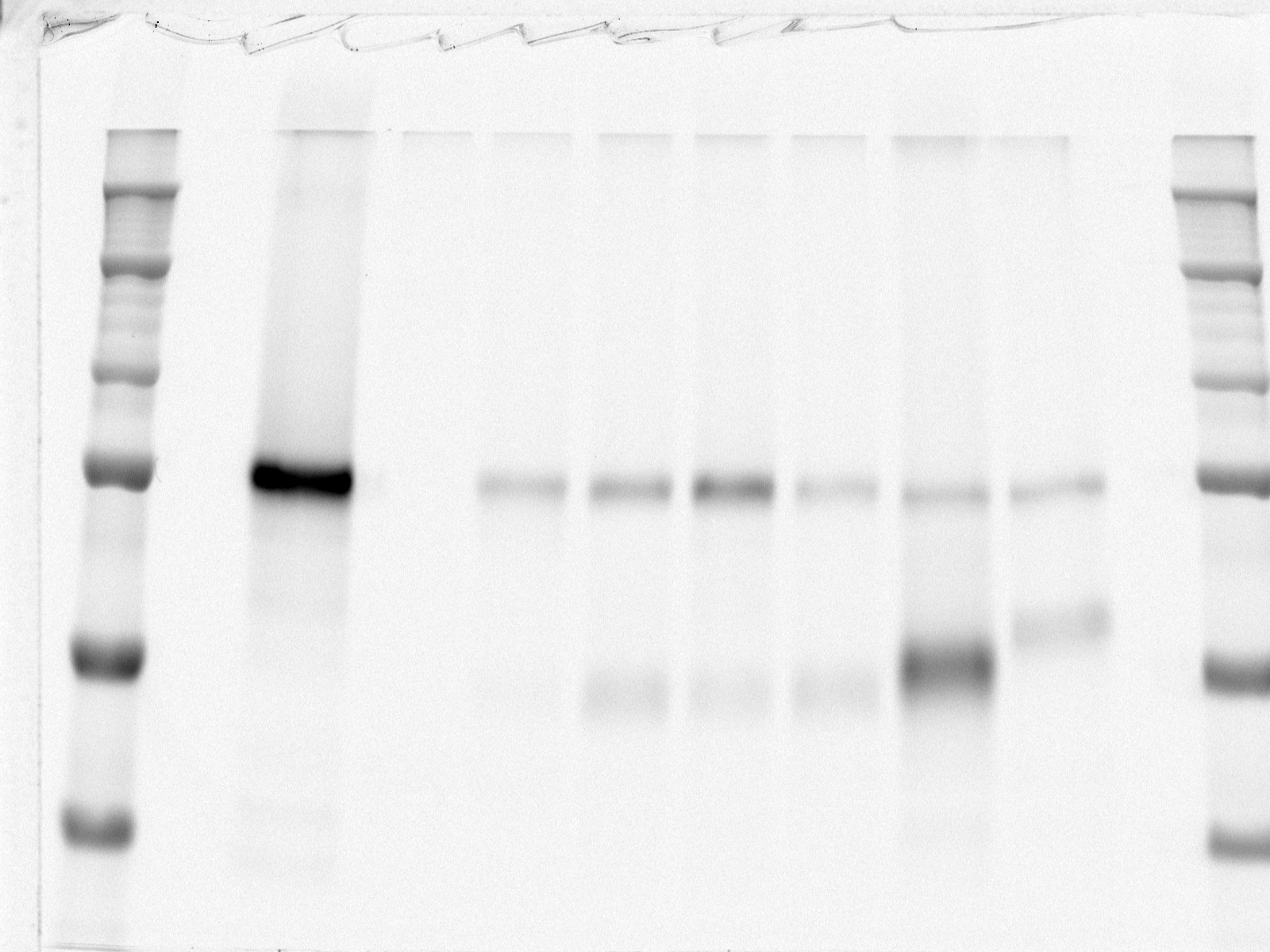

Supplement: Figure 2—source data 3. [file elife-82479-fig2-data3.zip › Figure-2_source-data-3/Gel8.gel]

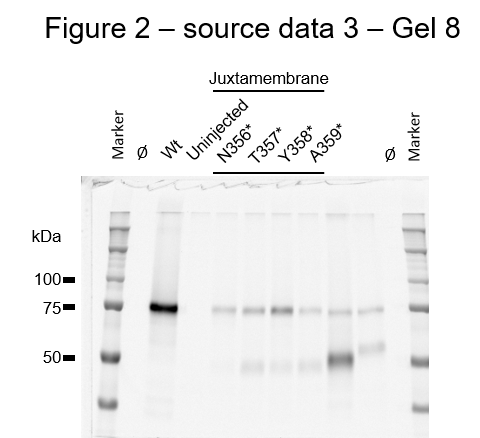

Supplement: Figure 2—source data 3. [file elife-82479-fig2-data3.zip › Figure-2_source-data-3/Gel8uncropped.png]

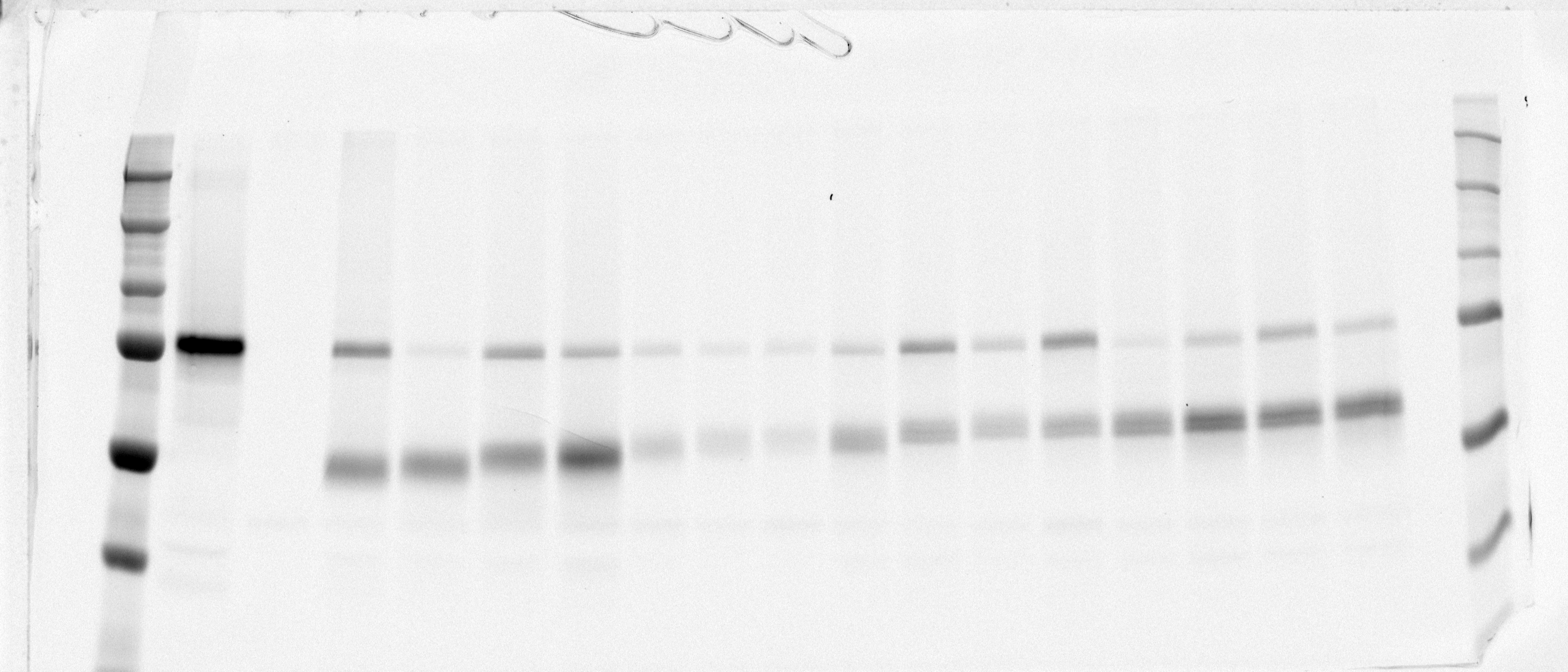

Supplement: Figure 2—source data 4. [file elife-82479-fig2-data4.zip › Figure-2_source-data-4/Gel9.gel]

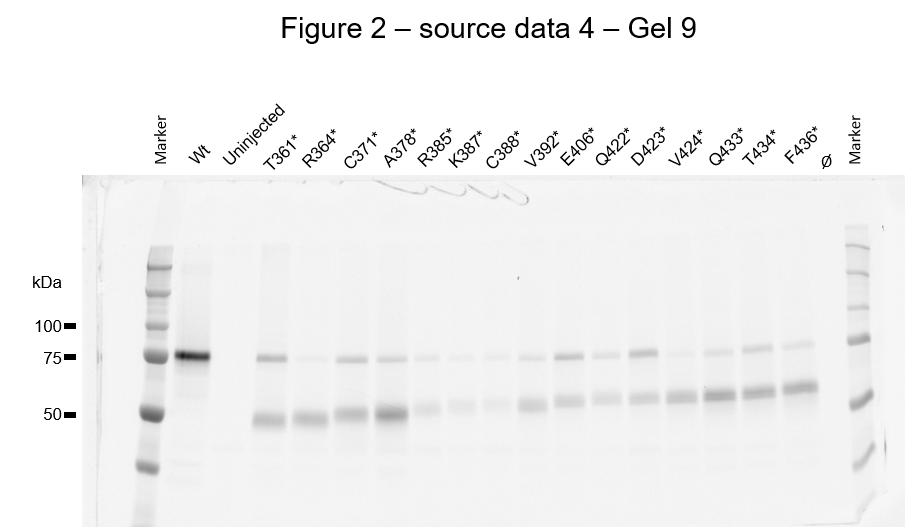

Supplement: Figure 2—source data 4. [file elife-82479-fig2-data4.zip › Figure-2_source-data-4/Gel9uncropped.png]

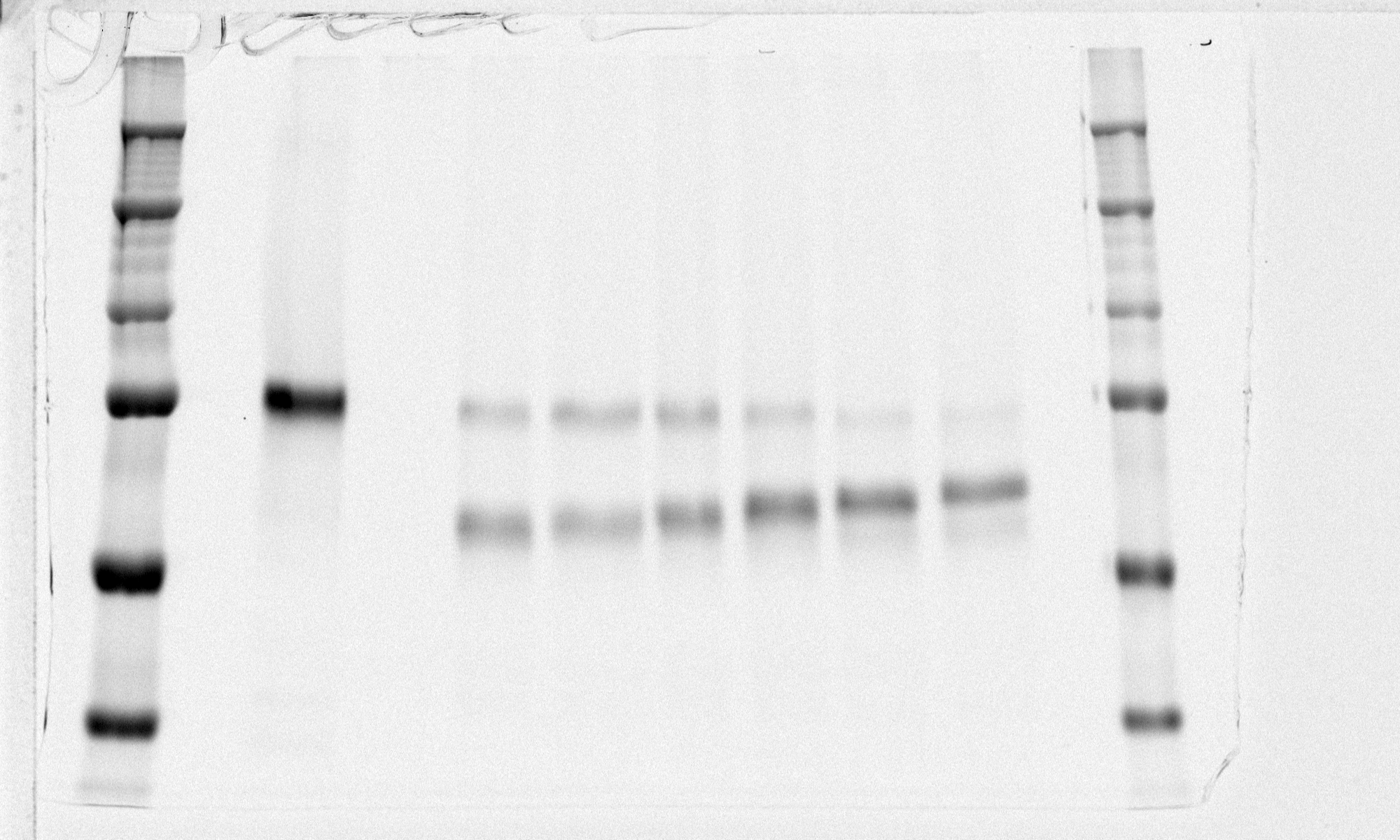

Supplement: Figure 2—source data 5. [file elife-82479-fig2-data5.zip › Figure-2_source-data-5/Gel10.gel]

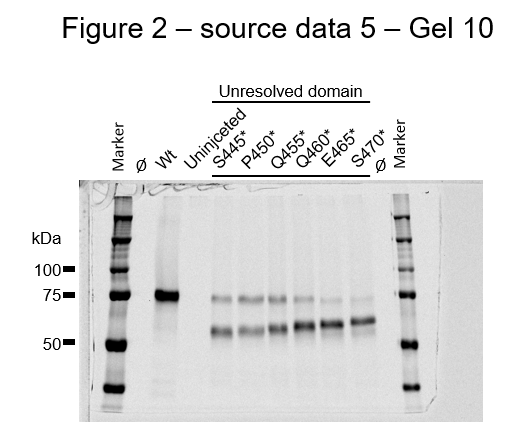

Supplement: Figure 2—source data 5. [file elife-82479-fig2-data5.zip › Figure-2_source-data-5/Gel10uncropped.png]

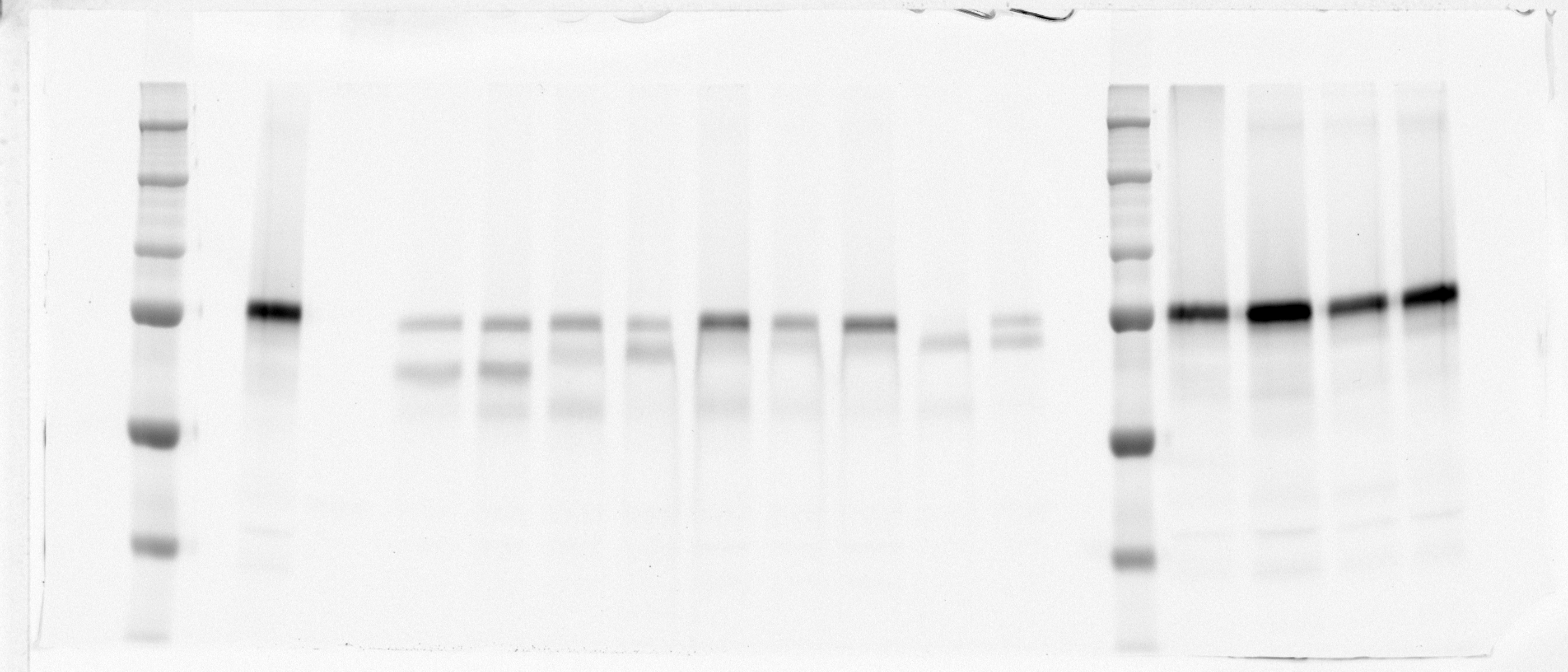

Supplement: Figure 2—source data 6. [file elife-82479-fig2-data6.zip › Figure-2_source-data-6/Gel11.gel]

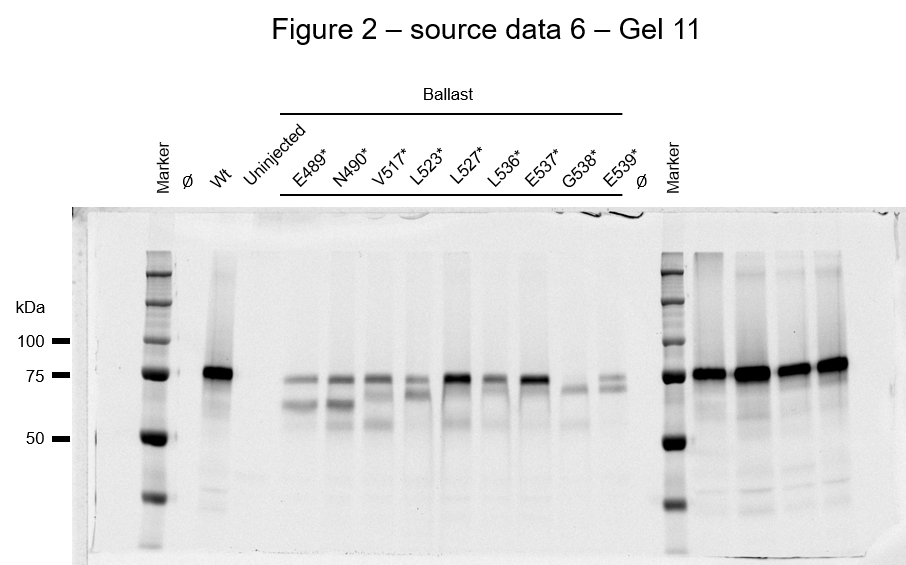

Supplement: Figure 2—source data 6. [file elife-82479-fig2-data6.zip › Figure-2_source-data-6/Gel11uncropped.png]

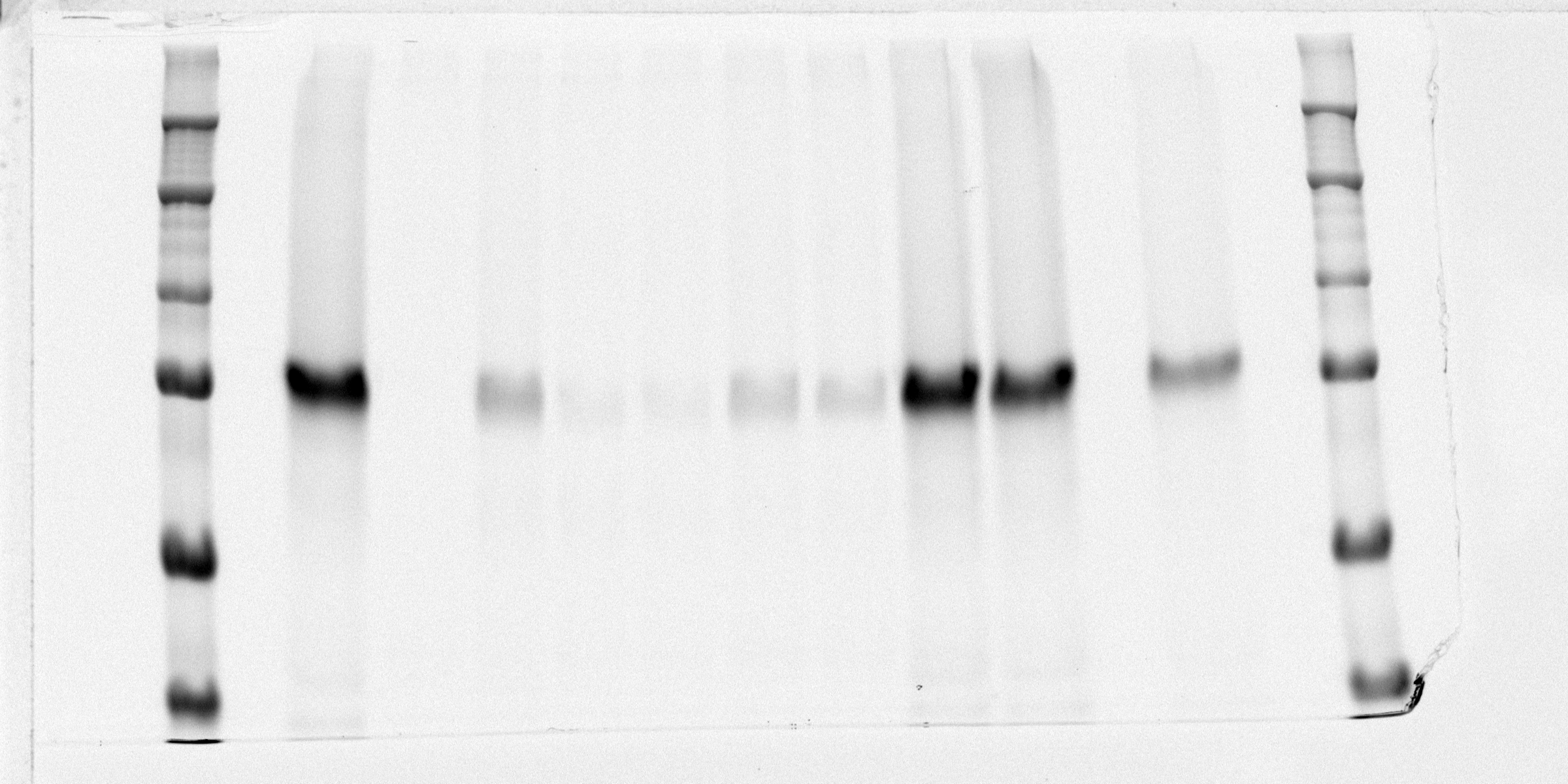

Supplement: Figure 2—source data 7. [file elife-82479-fig2-data7.zip › Figure-2_source-data-7/Gel12.gel]

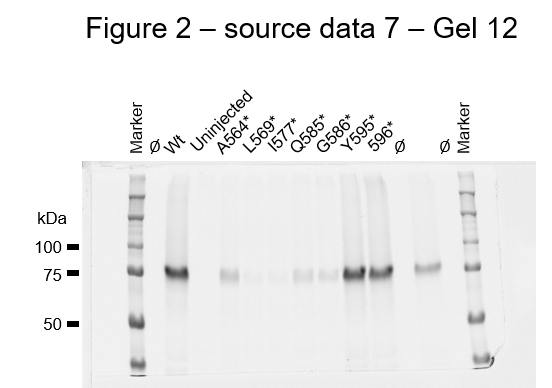

Supplement: Figure 2—source data 7. [file elife-82479-fig2-data7.zip › Figure-2_source-data-7/Gel12uncropped.png]

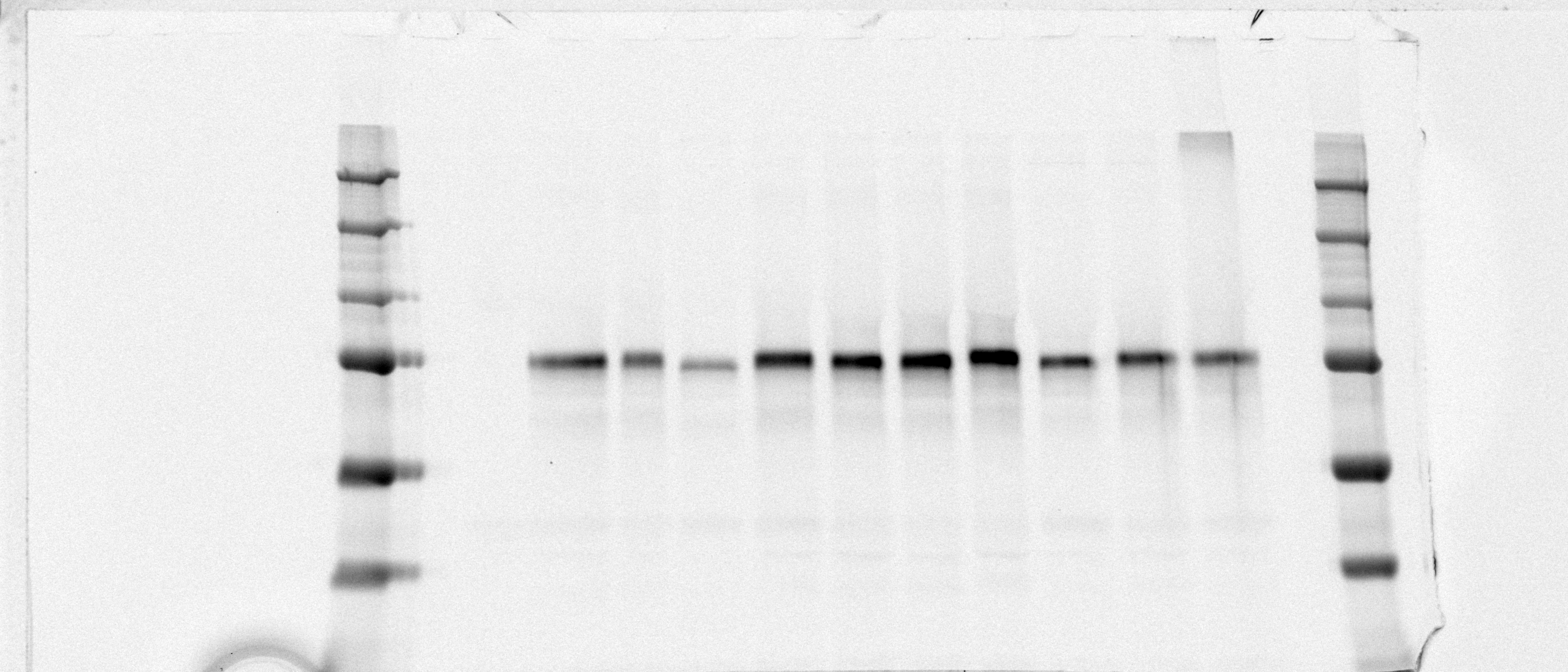

Supplement: Figure 2—figure supplement 1—source data 1. [file elife-82479-fig2-figsupp1-data1.zip › Figure-2_figure-supplement-1_source-data-1/Gel13_633nm.gel]

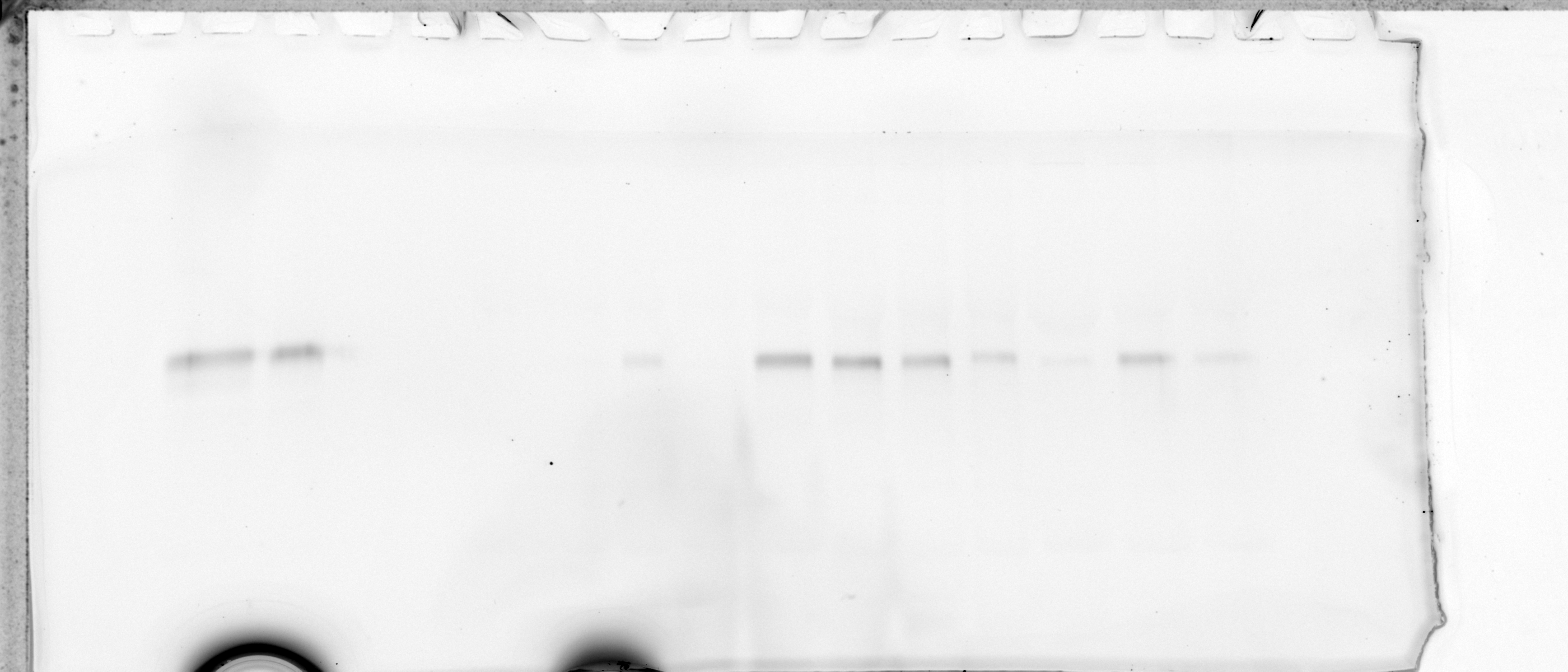

Supplement: Figure 2—figure supplement 1—source data 1. [file elife-82479-fig2-figsupp1-data1.zip › Figure-2_figure-supplement-1_source-data-1/Gel13_532nm.gel]

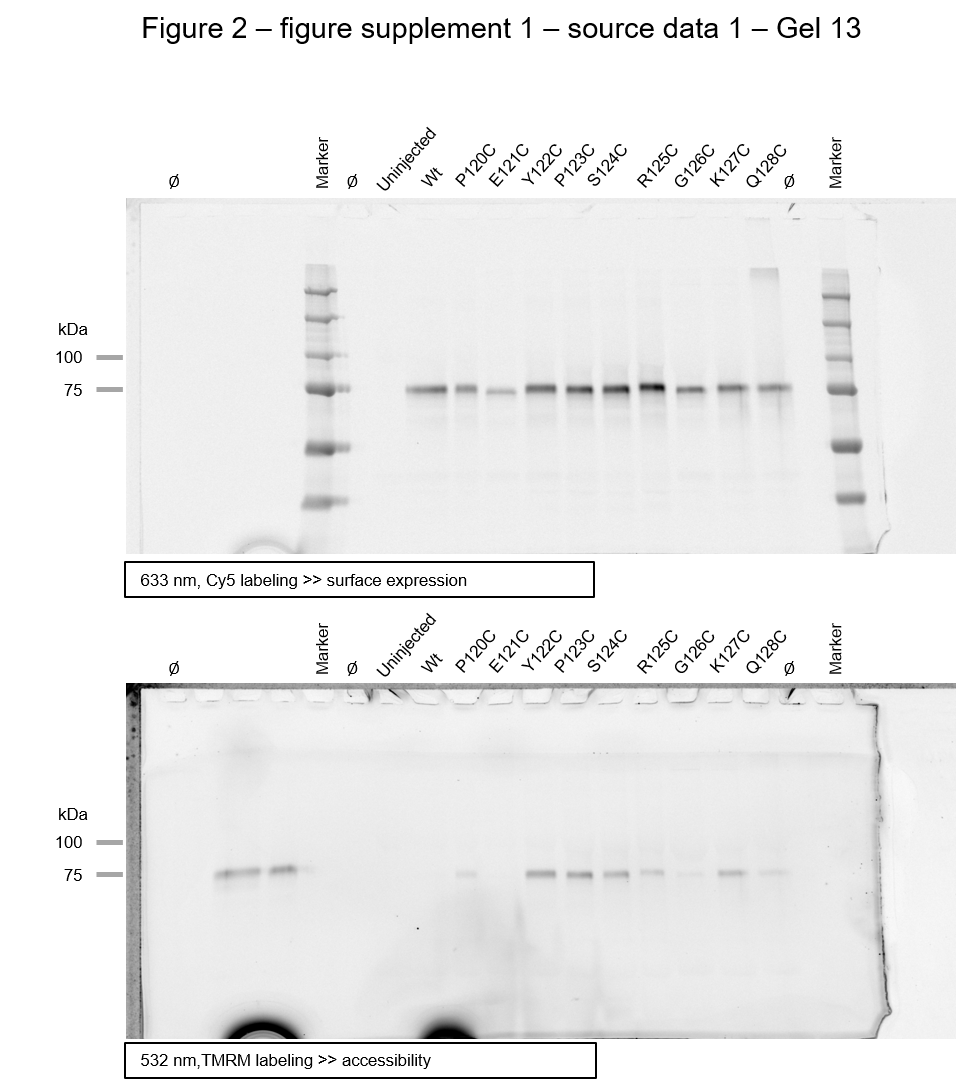

Supplement: Figure 2—figure supplement 1—source data 1. [file elife-82479-fig2-figsupp1-data1.zip › Figure-2_figure-supplement-1_source-data-1/Gel13uncropped.png]

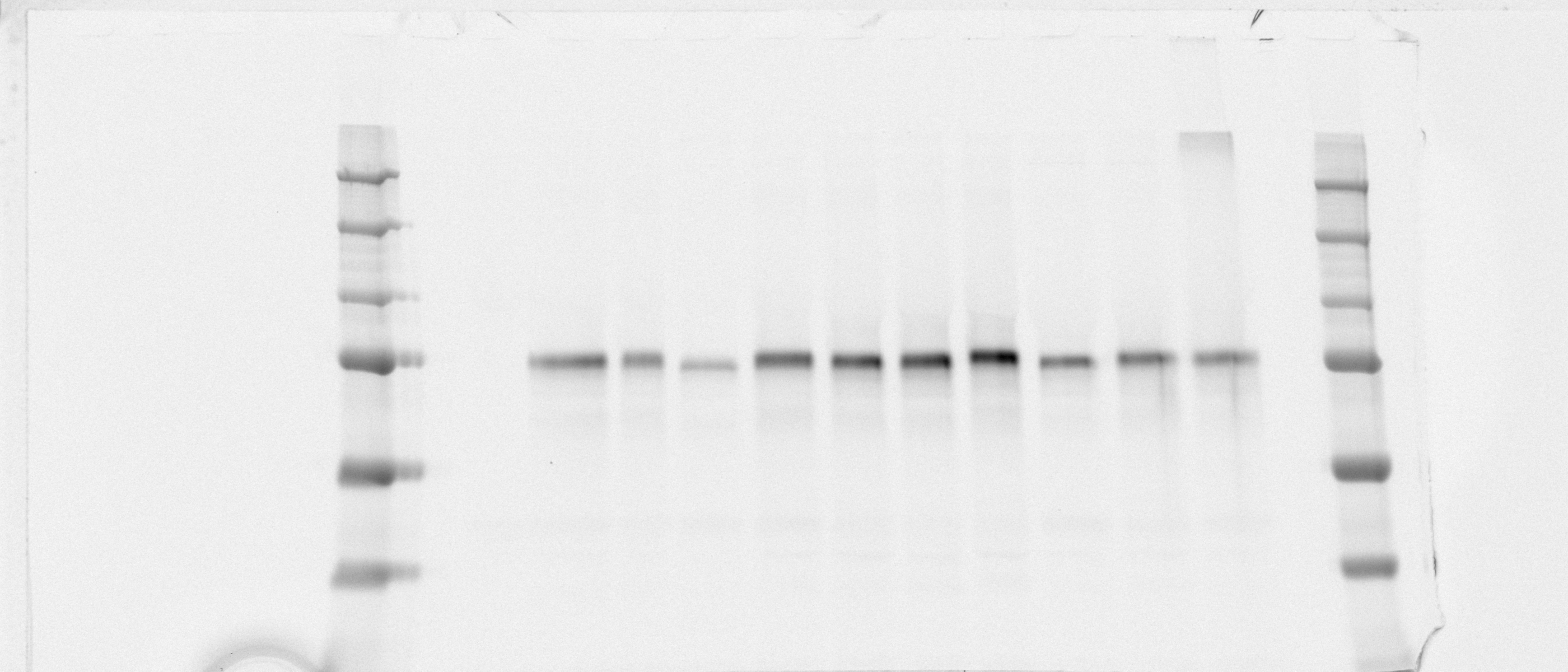

Supplement: Figure 2—figure supplement 1—source data 1. [file elife-82479-fig2-figsupp1-data1.zip › Figure-2_figure-supplement-1_source-data-1/Gel13_633nm.png]

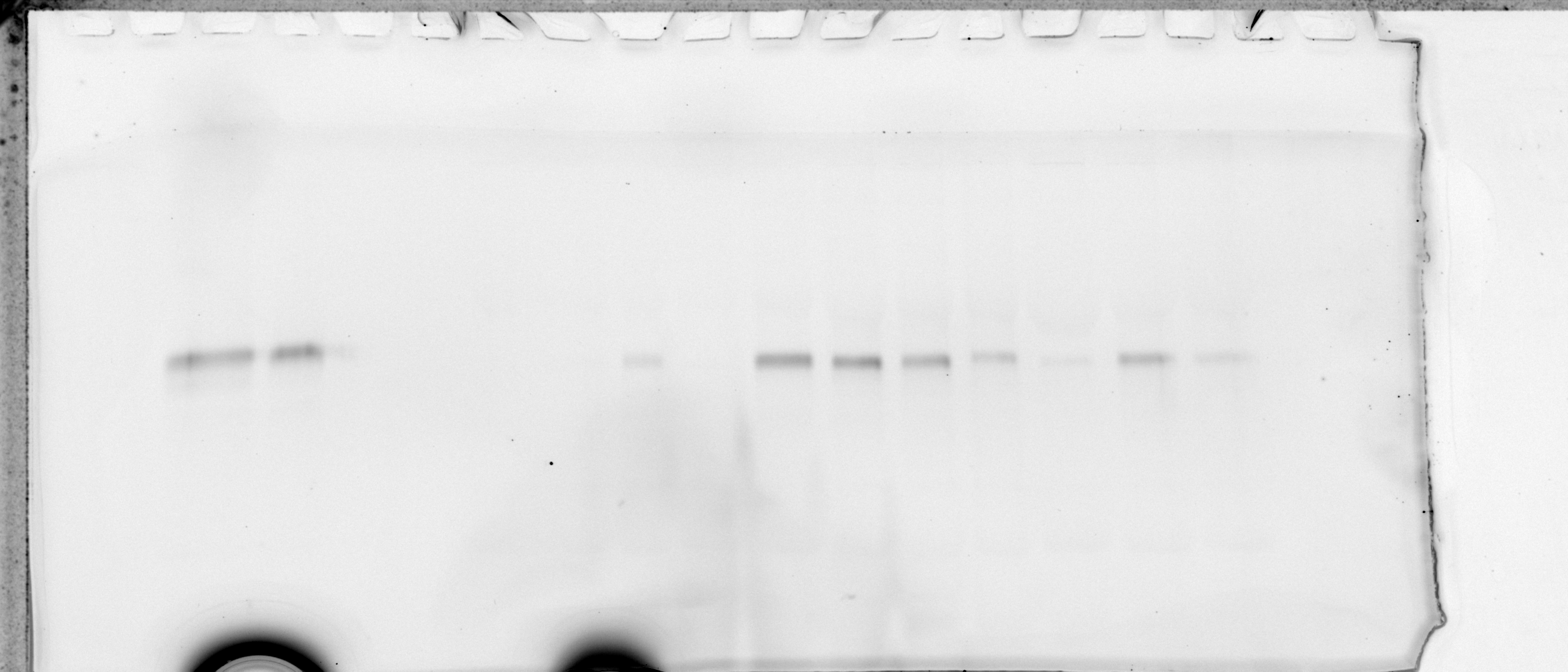

Supplement: Figure 2—figure supplement 1—source data 1. [file elife-82479-fig2-figsupp1-data1.zip › Figure-2_figure-supplement-1_source-data-1/Gel13_532nm.png]

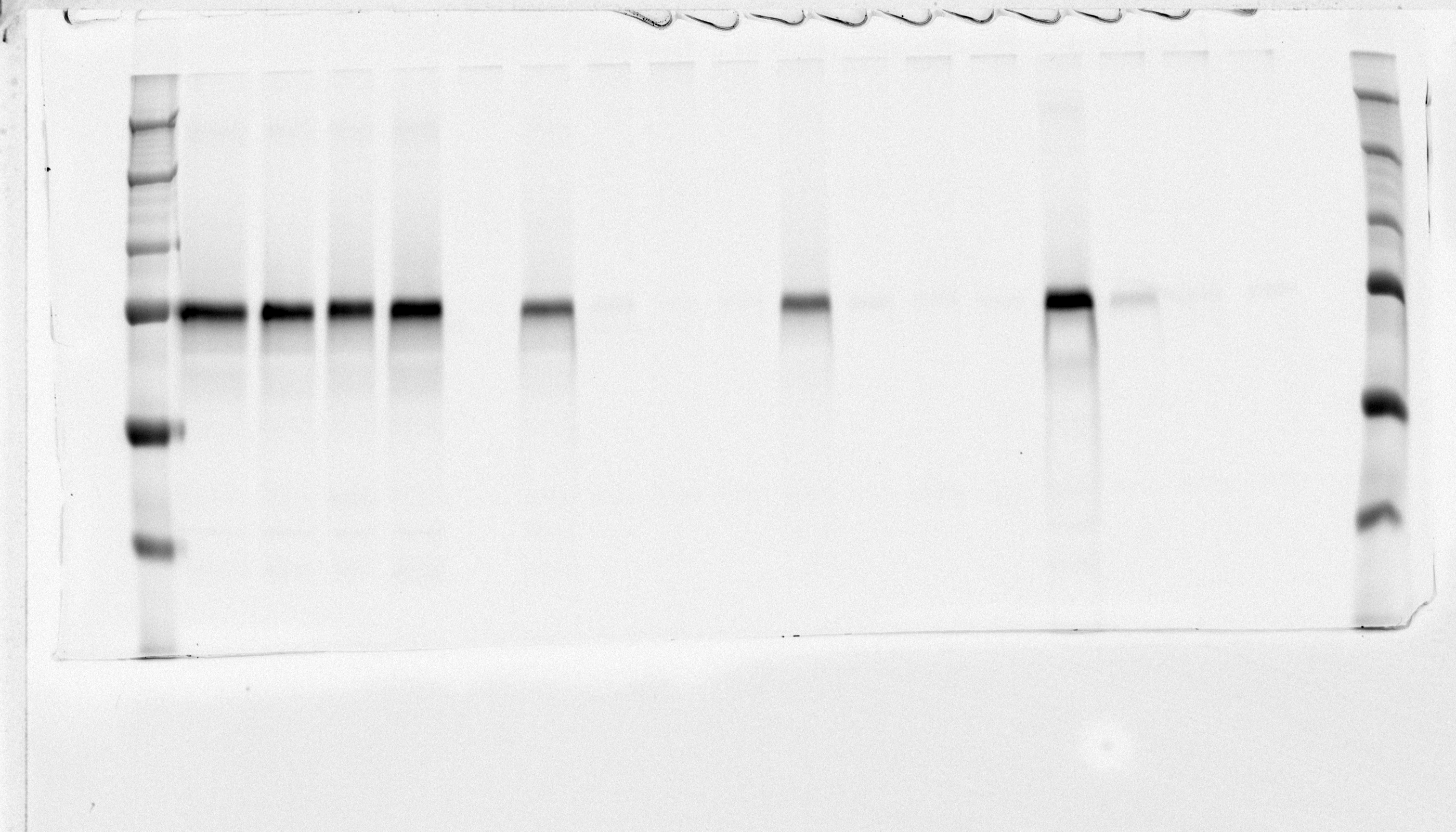

Supplement: Figure 3—figure supplement 2—source data 2. [file elife-82479-fig3-figsupp2-data2.zip › Figure-3_figure-supplement-2_source-data-2/Gel14.gel]

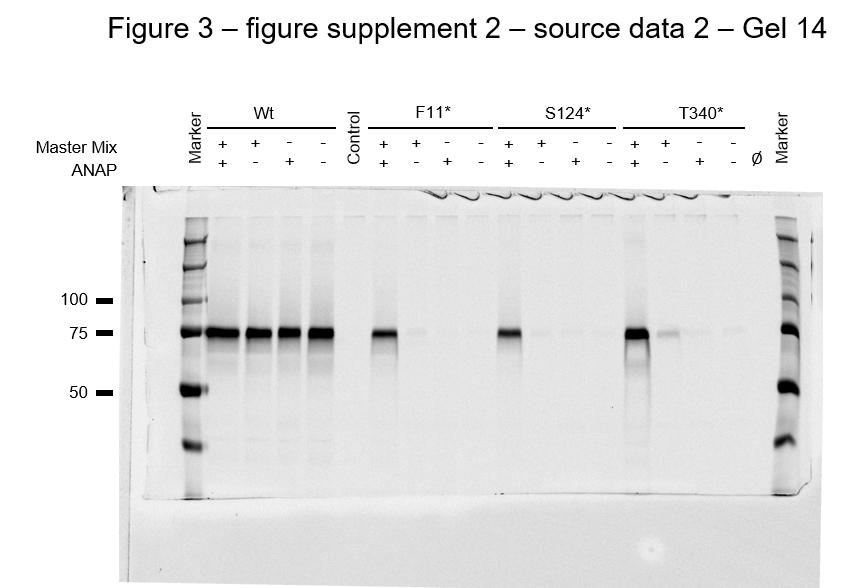

Supplement: Figure 3—figure supplement 2—source data 2. [file elife-82479-fig3-figsupp2-data2.zip › Figure-3_figure-supplement-2_source-data-2/Gel14_uncropped.png]

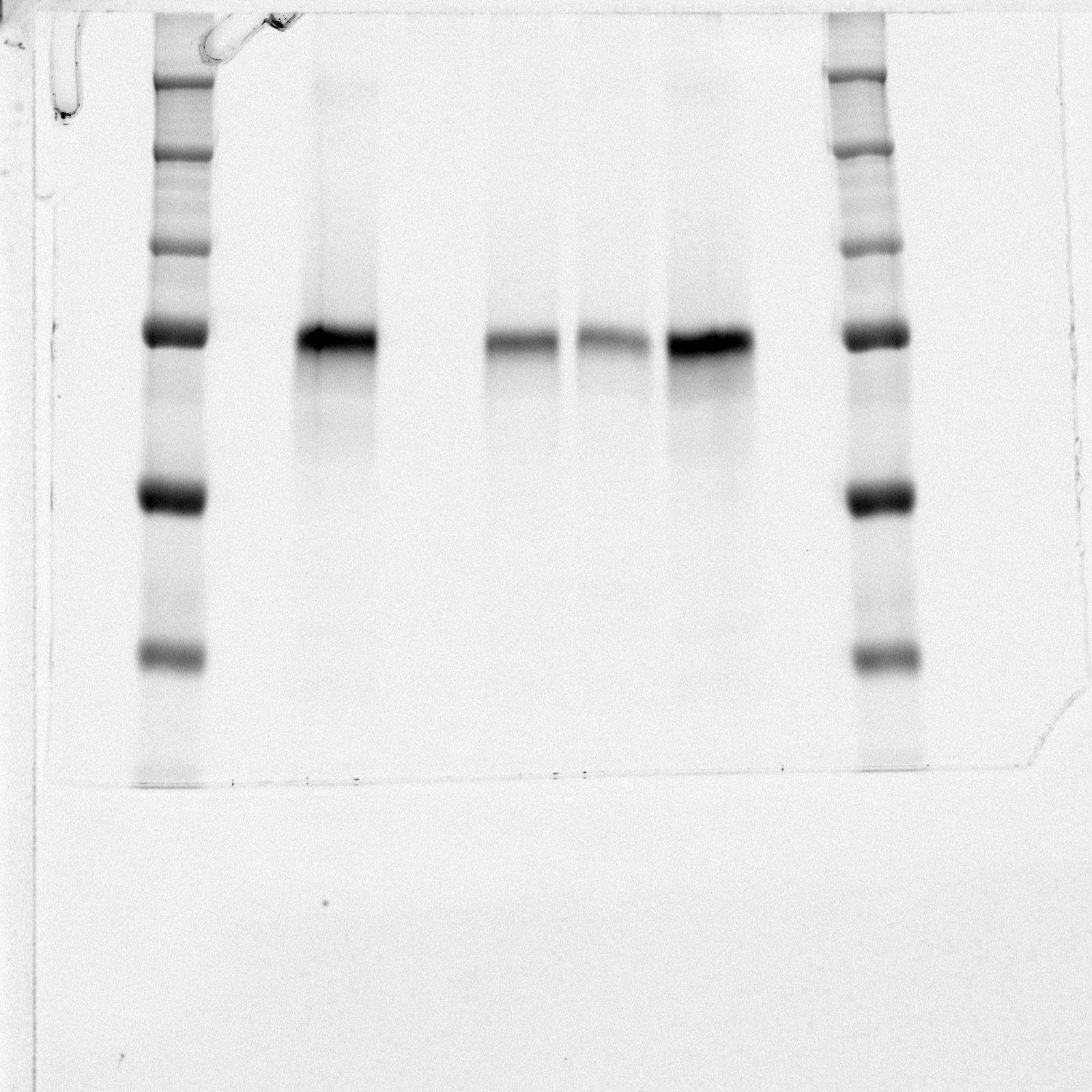

Supplement: Figure 5—figure supplement 1—source data 3. [file elife-82479-fig5-figsupp1-data3.zip › Figure-5_figure-supplement-1_source-data-3/Gel15.gel]

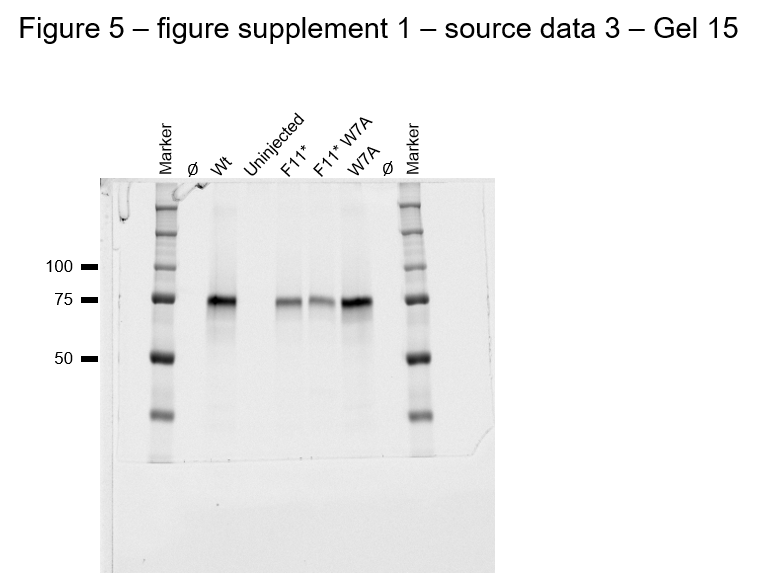

Supplement: Figure 5—figure supplement 1—source data 3. [file elife-82479-fig5-figsupp1-data3.zip › Figure-5_figure-supplement-1_source-data-3/Gel15uncropped.png]
