# Supplementary material for: Improved ANAP incorporation and VCF analysis reveal details of P2X7 current facilitation and a limited conformational interplay between ATP binding and the intracellular ballast domain
Source: eLife. 2023 Jan 4;12:e82479. doi: 10.7554/eLife.82479 (PMC9859053; doi:10.7554/eLife.82479)
Supplement: MDAR checklist [file elife-82479-mdarchecklist1.docx]

**Materials Design Analysis Reporting (MDAR)**

**Checklist for Authors**

The [MDAR framework](https://osf.io/xfpn4/) establishes a minimum set of requirements in transparent reporting mainly applicable to studies in the life sciences.

*eLife* asks authors to **provide detailed information within their article** to facilitate the interpretation and replication of their work. Authors can also upload supporting materials to comply with relevant reporting guidelines for health-related research (see [EQUATOR Network](http://www.equator-network.org/%20)), life science research (see the [BioSharing Information Resource](http://biosharing.org/)), or animal research (see the [ARRIVE Guidelines](http://www.plosbiology.org/article/info:doi/10.1371/journal.pbio.1000412) and the [STRANGE Framework](https://doi.org/10.1038/d41586-020-01751-5); for details, see *eLife*’s [Journal Policies](https://reviewer.elifesciences.org/author-guide/journal-policies)). Where applicable, authors should refer to any relevant reporting standards materials in this form.

For all that apply, please note **where in the article** the information is provided. Please note that we also collect information about data availability and ethics in the submission form.

**Materials:**

| **Newly created materials** | **Indicate where provided: section/figure legend** | **N/A** |
| --- | --- | --- |
| The manuscript includes a dedicated "materials availability statement" providing transparent disclosure about availability of newly created materials including details on how materials can be accessed and describing any restrictions on access. | Sequences of newly designed cDNA or recombinant DNA are detailed in the Key Resource Table and Material and Methods section and the respective plasmids will be made available upon request. |  |
|  |  |  |
| **Antibodies** | **Indicate where provided: section/figure legend** | **N/A** |
| For commercial reagents, provide supplier name, catalogue number and [RRID](https://scicrunch.org/resources), if available. | These details are provided in the Key Resource Table and Material and Methods section |  |
|  |  |  |
| **DNA and RNA sequences** | **Indicate where provided: section/figure legend** | **N/A** |
| Short novel DNA or RNA including primers, probes: Sequences should be included or deposited in a public repository. | This information is given in the Key Resource Table and in the Material and Methods section ‘cDNA and cloning’. |  |
|  |  |  |
| **Cell materials** | **Indicate where provided: section/figure legend** | **N/A** |
| Cell lines: Provide species information, strain. Provide accession number in repository OR supplier name, catalog number, clone number, OR RRID. | Not used in this study | N/A |
| Primary cultures: Provide species, strain, sex of origin, genetic modification status. | Not used in this study | N/A |
|  |  |  |
| **Experimental animals** | **Indicate where provided: section/figure legend** | **N/A** |
| Laboratory animals or Model organisms: Provide species, strain, sex, age, genetic modification status. Provide accession number in repository OR supplier name, catalog number, clone number, OR RRID. | The sources of *Xenopus laevis* oocytes (*Xenopus laevis* females from Nasco International Cat#:LM00535 and Ecocyte Biosciences) are given in Material and Methods. |  |
| Animal observed in or captured from the field: Provide species, sex, and age where possible. | Not used | N/A |
|  |  |  |
| **Plants and microbes** | **Indicate where provided: section/figure legend** | **N/A** |
| Plants: provide species and strain, ecotype and cultivar where relevant, unique accession number if available, and source (including location for collected wild specimens). | Not used | N/A |
| Microbes: provide species and strain, unique accession number if available, and source. | Not used | N/A |
|  |  |  |
| **Human research participants** | **Indicate where provided: section/figure legend) or state if these demographics were not collected** | **N/A** |
| If collected and within the bounds of privacy constraints report on age, sex, gender and ethnicity for all study participants. | Not applicable | **N/A** |

**Design:**

| **Study protocol** | **Indicate where provided: section/figure legend** | **N/A** |
| --- | --- | --- |
| If the study protocol has been pre-registered, provide DOI. For clinical trials, provide the trial registration number OR cite DOI. | Not applicable | N/A |
|  |  |  |
| **Laboratory protocol** | **Indicate where provided: section/figure legend** | **N/A** |
| Provide DOI OR other citation details if detailed step-by-step protocols are available. | DOI: 10.1007/978-1-0716-2384-8_10 |  |
|  |  |  |
| **Experimental study design (statistics details) *** | | |
| **For in vivo studies: State whether and how the following have been done** | **Indicate where provided: section/figure legend. If it could have been done, but was not, write “not done”** | **N/A** |
| Sample size determination | No *in vivo* studies were performed | N/A |
| Randomisation | No *in vivo* studies were performed | N/A |
| Blinding | No *in vivo* studies were performed | N/A |
| Inclusion/exclusion criteria | No *in vivo* studies were performed | N/A |
|  |  |  |
| **Sample definition and in-laboratory replication** | **Indicate where provided: section/figure legend** | **N/A** |
| State number of times the experiment was replicated in the laboratory. | SDS-PAGE analyses were replicated in at least two independent experiments as specified in figure legend 1 B and D, and 2 C.  For each construct and filterset, 3-50 VCF recordings were performed (criteria for successful recordings are specified in table legend 1 and Material and Methods).  VCF-measurements for which example recordings are shown, were replicated at least three times. The actual numbers are shown in brackets in Fig. 4 B and C, 5 B and D. In case of overlay representations, numbers are given in figure legends 3 F, 4 D, 5 C, and 6 A. |  |
| Define whether data describe technical or biological replicates. | Experiments were replicated with *Xenopus laevis* oocytes from at least two different animals (≥ two biological replicates). |  |
|  |  |  |
| **Ethics** | **Indicate where provided: section/submission form** | **N/A** |
| Studies involving human participants: State details of authority granting ethics approval (IRB or equivalent committee(s), provide reference number for approval. |  | N/A |
| Studies involving experimental animals: State details of authority granting ethics approval (IRB or equivalent committee(s), provide reference number for approval. | *Xenopus laevis* females were kept  at the Core Facility Animal Models (CAM) of the Biomedical Center (BMC) of LMU Munich, Germany  (Az:4.3.2–5682/LMU/BMC/CAM) |  |
| Studies involving specimen and field samples: State if relevant permits obtained, provide details of authority approving study; if none were required, explain why. |  | N/A |
|  |  |  |
| **Dual Use Research of Concern (DURC)** | **Indicate where provided: section/submission form** | **N/A** |
| If study is subject to dual use research of concern regulations, state the authority granting approval and reference number for the regulatory approval. |  | N/A |

**Analysis:**

| **Attrition** | **Indicate where provided: section/figure legend** | **N/A** |
| --- | --- | --- |
| Describe whether exclusion criteria were pre-established. Report if sample or data points were omitted from analysis. If yes, report if this was due to attrition or intentional exclusion and provide justification. | Exclusion criteria for VCF recordings (described in Material and Methods/Data analysis):  1) drifting leak current (> 0.1 μA / 10 sec)  2) current recordings < 0.1 μA or not reproducible  3) fluorescence recordings not reproducible (in less than three different oocytes and less than 40% of measured oocytes) and averaged ΔF/F values < 0.3%.  4) fluorescence recordings not differentiable from wt-expressing oocytes. In this case, ΔF/F was defined as 0% (Fig. 3 D, 4 B and C, 5 B and D) and no statistical analysis was performed. |  |
|  |  |  |
| **Statistics** | **Indicate where provided: section/figure legend** | **N/A** |
| Describe statistical tests used and justify choice of tests. | The two-tailed unpaired Welch’s t-test and the two-tailed paired Student’s t-test were used for statistical analysis, as specified in the figure legends of Fig. 1 D, Fig. 4 B, and Fig. 3-Fig.Suppl.3C, Fig.4-Fig.Suppl.1B, Fig.5-Fig.Suppl.3B, respectively, and in the Material and Methods section ‘Statistical Analysis’. |  |
|  |  |  |
| **Data availability** | **Indicate where provided: section/submission form** | **N/A** |
| For newly created and reused datasets, the manuscript includes a data availability statement that provides details for access (or notes restrictions on access). | Source data are provided for each figure, table, and figure supplement. | N/A |
| When newly created datasets are publicly available, provide accession number in repository OR DOI and licensing details where available. | Not applicable. | N/A |
| If reused data is publicly available provide accession number in repository OR DOI, OR URL, OR citation. | PDB ID: 6u9w  McCarthy, A.E., Yoshioka, C., Mansoor, S.E., 2019. Full-Length P2X7 Structures Reveal How Palmitoylation Prevents Channel Desensitization. Cell 179, 659-670.e13. https://doi.org/10.1016/j.cell.2019.09.017 | N/A |
|  |  |  |
| **Code availability** | **Indicate where provided: section/figure legend** | **N/A** |
| For any computer code/software/mathematical algorithms essential for replicating the main findings of the study, whether newly generated or re-used, the manuscript includes a data availability statement that provides details for access or notes restrictions. | No new computer code, software, or mathematical algorithms essential for replicating the main findings of the study were used. Information concerning analysis using either specific software or a Python-based script is provided in Material and Methods / Data analysis and in the Key Resource Table. | N/A |
| Where newly generated code is publicly available, provide accession number in repository, OR DOI OR URL and licensing details where available. State any restrictions on code availability or accessibility. | Not applicable. | N/A |
| If reused code is publicly available provide accession number in repository OR DOI OR URL, OR citation. | Information regarding software used in this study is given in Material and Methods / Data analysis and in the Key Resource Table. | N/A |

**Reporting:**

The MDAR framework recommends adoption of discipline-specific guidelines, established and endorsed through community initiatives.

| **Adherence to community standards** | **Indicate where provided: section/figure legend** | **N/A** |
| --- | --- | --- |
| State if relevant guidelines (e.g., ICMJE, MIBBI, ARRIVE, STRANGE) have been followed, and whether a checklist (e.g., CONSORT, PRISMA, ARRIVE) is provided with the manuscript. |  | N/A |

* We provide the following guidance regarding transparent reporting and statistics; we also refer authors to [Ten common statistical mistakes to watch out for when writing or reviewing a manuscript](https://doi.org/10.7554/eLife.48175).

**Sample-size estimation**

- You should state whether an appropriate sample size was computed when the study was being designed
- You should state the statistical method of sample size computation and any required assumptions
- If no explicit power analysis was used, you should describe how you decided what sample (replicate) size (number) to use

**Replicates**

- You should report how often each experiment was performed
- You should include a definition of biological versus technical replication
- The data obtained should be provided and sufficient information should be provided to indicate the number of independent biological and/or technical replicates
- If you encountered any outliers, you should describe how these were handled
- Criteria for exclusion/inclusion of data should be clearly stated
- High-throughput sequence data should be uploaded before submission, with a private link for reviewers provided (these are available from both GEO and ArrayExpress)

**Statistical reporting**

- Statistical analysis methods should be described and justified
- Raw data should be presented in figures whenever informative to do so (typically when N per group is less than 10)
- For each experiment, you should identify the statistical tests used, exact values of N, definitions of center, methods of multiple test correction, and dispersion and precision measures (e.g., mean, median, SD, SEM, confidence intervals; and, for the major substantive results, a measure of effect size (e.g., Pearson's r, Cohen's d)
- Report exact p-values wherever possible alongside the summary statistics and 95% confidence intervals. These should be reported for all key questions and not only when the p-value is less than 0.05.

**Group allocation**

- Indicate how samples were allocated into experimental groups (in the case of clinical studies, please specify allocation to treatment method); if randomization was used, please also state if restricted randomization was applied
- Indicate if masking was used during group allocation, data collection and/or data analysis
